# Audience effect on domestic dogs’ behavioural displays and facial expressions

**DOI:** 10.1038/s41598-022-13566-7

**Published:** 2022-06-13

**Authors:** Giulia Pedretti, Chiara Canori, Sarah Marshall-Pescini, Rupert Palme, Annalisa Pelosi, Paola Valsecchi

**Affiliations:** 1 Department of Medicine and Surgery, University of Parma, Via Gramsci 14, 43126 Parma, Italy; 2 Department of Chemistry, Life Science and Environmental Sustainability, University of Parma, Viale delle Scienze 17/A, 43124 Parma, Italy; 3 Domestication Lab, Wolf Science Center, Konrad-Lorenz-Institute for Ethology, University of Veterinary Medicine, Veterinärplatz 1, 1210 Vienna, Austria; 4 Unit of Physiology, Pathophysiology and Experimental Endocrinology, Department of Biomedical Sciences, University of Veterinary Medicine, Veterinärplatz 1, 1210 Vienna, Austria

**Keywords:** Emotion, Social behaviour, Stress and resilience, Animal behaviour, Animal physiology

## Abstract

In the present study we investigated the influence of positive and negative arousal situations and the presence of an audience on dogs’ behavioural displays and facial expressions. We exposed dogs to positive anticipation, non-social frustration and social frustration evoking test sessions and measured pre and post-test salivary cortisol concentrations. Cortisol concentration did not increase during the tests and there was no difference in pre or post-test concentrations in the different test conditions, excluding a different level of arousal. Displacement behaviours of “looking away” and “sniffing the environment” occurred more in the frustration-evoking situations compared to the positive anticipation and were correlated with cortisol concentrations. “Ears forward” occurred more in the positive anticipation condition compared to the frustration-evoking conditions, was positively influenced by the presence of an audience, and negatively correlated to the pre-test cortisol concentrations, suggesting it may be a good indicator of dogs’ level of attention. “Ears flattener”, “blink”, “nose lick”, “tail wagging” and “whining” were associated with the presence of an audience but were not correlated to cortisol concentrations, suggesting a communicative component of these visual displays. These findings are a first step to systematically test which subtle cues could be considered communicative signals in domestic dogs.

## Introduction

Behavioural patterns may function as communicative signals, in which case they are referred to as ‘displays’^1^. Communication occurs when the actions of one individual, the sender, provides a signal that changes the distribution of the probability of the behaviour of another individual, the receiver^2^. Many displays consist of behaviours that originally did not have a communicative function (e.g. autonomic or protective responses) but evolved to become efficient signals^3^. Because behavioural patterns may arise from generalized excitement and/or stress (physiological reaction of the organism) it is not always easy to understand whether they include a communicative function. Animals can transmit signals in an active or more passive/involuntary manner. A criterion that allows to disentangle behavioural patterns caused by generalized excitement (physiological reaction of the organism) from those actively performed as means of communication is to observe their dependence on the presence of a social partner, the so-called “audience effect”^4^. Although whether “theory of mind” (understanding of the social partner’s perspective) and signaller intentionality (being deliberate or purposive) are involved in such processes is still a hotly debated issue (see^5,6^).

Displays can originate from different behavioural categories including facial expressions, and displacement behaviours^7^. Facial expressions are movements of the muscles of the facial regions and have been considered potential indicators of different affective states^8^ in both humans^9,10^ and other animals^11,12^. Facial expression components could have evolved as signals in social interactions by providing the receiver with information about the senders’ affective state (as suggested by the behavioural ecology view of facial displays^13^) and hence inform the receiver of the sender’s motivations and potential future actions^14,15^. Signaling distress or frustration could benefit the sender as well as the receiver in a social context since proper reaction to, for example, fear-related expressions could contribute to an individual’s survival^16^ favoring the evolution of such behaviours. In line with this, some evidence shows that the production of primates’ facial expressions as well as other behavioural displays (e.g. gestures) are dependent on the presence of an audience^17,18^, suggesting that they can be flexibly used as visual signals by the senders.

Displacement behaviours are behavioural patterns displayed outside their motivational context and without a direct functionality relating to the ongoing situation^3^. They include acts of self-maintenance such as self-grooming, scratching, yawning, lips licking^19^ but also environment-directed behaviours such as sniffing or manipulation of objects in the environment^20^. Displacement behaviours have been identified as indicators of frustration and motivational conflict^21,22^ and a by-product of the physiological stress response^19^. In fact, they could function as coping strategies, to restore the individual’s homeostasis after exposure to a stressor^21^. Displacement behaviours have also been linked to social anxiety in primates with their occurrence being higher in socially unstable communities compared to more stable ones^23,24^. It has been hypothesized that, since displacement behaviours are usually very evident (e.g. scratching, yawning, self-grooming), they could provide some information to others, and thus have a communicative function. This hypothesis has only been tested in primates showing that the production of self-directed behaviours can affect the immediate behaviours of others, thus having an adaptational value for the sender and reducing the probability of aggressive interactions^25,26^.

In domestic dogs, previous research has identified several behavioural patterns and facial expressions as being indicative of negative affective states. Regarding facial expressions, backwards directed ears, tongue out and showing the sclera (“whale eye”) have been observed in a fear-related context (fireworks exposition^27^) while flattened ears, lips part, jaw drop and the facial displacement behaviours of lips/nose lick and blink have been linked to frustration-evoking situations^28,29^. However, lips licking and nose lick have also been associated with positive anticipation situations, suggesting a link to general arousal (not specifically carrying a negative valence)^30^.

Different displacement behaviours are elicited in dogs by noxious stimuli or restricted housing conditions: yawning^31^, paw-lifting^32^, stretching^31^, auto-grooming^33^, manipulation, sniffing of the environment^34,35^ and looking away^36^^.^ The link between these sets of behaviours and stressful situations is well supported by observational evidence, however there is no standardization in methodology of testing and behavioural definition. Furthermore, only few studies have investigated the physiological correlates of these sets of behaviours, with mixed results.

For example, after ‘aversive training’ dogs show increased saliva cortisol (a glucocorticoid linked to the stress response, specifically the activation of HPA axis—hypothalamic–pituitary–adrenal axis^37^) and a higher frequency of lips licking, yawning, and body shaking in comparison to dogs that experienced ‘positive training’ sessions^38^. However, other studies did not find a direct correlation between cortisol concentrations (measured in blood, urine, or saliva) and the performance of displacement behaviours in putatively stressful situations^39^. Beerda and colleagues^40^ found that dogs’ saliva cortisol increased just after non-social stressors (e.g. loud noise and electric shock) and positively correlated with general low posture but not with other displacement behaviours (e.g. nose lick, tongue out, shaking) that were only observed during stimuli administered by a human experimenter. Finally, some studies actually found a negative correlation between cortisol and displacement behaviours (digging and licking^35^, sniffing the environment^41^), suggesting a potential de-arousal function of these behaviours^42^.

The displacement behaviours cited above (looking away, blinking, licking nose/lips, yawning, sniffing the environment) have also been suggested to have a communicative intent and more specifically to function as appeasement signals in dog–dog and dog–human interactions^43^. Appeasement signals are behaviours that, by communicating the animal’s lack of agonistic motivation, should reduce the likelihood of aggressive behaviours from the receiver^31^. A few observational studies found lips-licking, looking away and yawning to be effective appeasement signals, decreasing the rate of aggression in groups of familiar and unfamiliar dogs^44,45^. Dogs were also noted to display the aforesaid signals during their interactions with humans^31,46^. However, whether the emission of such behaviours was due to the presence of the social partner (dog or human), or whether it was elicited by a more general physiological activation caused by the experimental situation has yet to be determined.

The audience effect on dogs’ facial and behavioural displays has been scarcely studied. In a conspecific play context dogs show sensitivity to the visual attention of their partners in that they exhibit play signals exclusively to forward-facing partners^47^. Kaminski and colleagues^48^ investigated whether domestic dog facial expressions were influenced by the attentional state of a human experimenter and/or a non-social arousing stimuli (food). While the presence of the food didn’t have any influence on the behaviour, the “inner brow raiser” and the “tongue show” were positively influenced by the attentional state of the experimenter. However, evidence is mixed^29^ and limited to only few behavioural and facial displays (Inner Brow Raiser, play signals, showing behaviour).

Our study aimed to assess, with a standardized experimental paradigm, whether dogs’ displacement behaviours and facial expressions are linked to a negative state of arousal (frustration), compared to a positive one (positive anticipation), and whether they are dependent to the presence of an audience (in our case an attentive human experimenter), thus carrying a basic communicative valence.

To achieve our goal, we adopted an experimental paradigm previously used to investigate differences in facial expressions during positive anticipation and frustration situations in dogs when waiting for a reward^28,29,49^^.^ The basic setup involves a dog seeing a piece of food placed on a table behind a plexiglass screen. After waiting a few seconds, the screen is removed allowing the dog to access the reward (positive anticipation). However, in the frustration condition, the food is removed, and dogs cannot obtain it. In the current modified version, we altered the conditions varying also the visibility of the audience (a person). Thus, we exposed dogs to 3 different test conditions: positive anticipation (positive arousal—no presence of an audience), frustration non-social (negative arousal—no presence of an audience) and frustration social (negative arousal—presence of an audience). To control for the influence of HPA axis activation that could vary between the conditions we collected salivary cortisol before and after each test.

Based on the current literature, we expected a higher likelihood of occurrence of behavioural indicators and facial expressions previously related to negative emotional states in the frustration evoking situation compared to the positive anticipation situation and we expected them to be associated with an increase in salivary cortisol concentration during the test. Furthermore, we expected a higher likelihood of occurrence of putative communicative signals when an audience was visible compared to when it was not.

## Results

Nine DogFACS variables and five general behaviour variables were included in the statistical analysis since they satisfied the selection criterion (units and behaviours observed in at least 10% of dogs in at least of one of the three test conditions). Self-directed displacement behaviours, like yawning, auto-grooming, scratching, stretching, shaking and environment-directed displacement behaviours like drinking and scratching the door were excluded from statistical analysis because they were expressed only by a few subjects. No dogs performed scratching, shaking, and drinking or scratching the door. One dog performed stretching in the frustration non-social condition. One dog performed auto-grooming in the frustration non-social and another dog in the frustration social condition. Yawning was performed by 2 dogs in the frustration non-social and by 4 dogs in the frustration social condition (Table 1).

**Table 1:** Behaviours selected for statistical analysis.

Ethogram	Category	Behaviour
DogFACS	Upper face Action Units (AUs)	Inner brow raiser (AU101)
		Blink (AU145)
	Mouth Action Descriptors (ADs)	Nose lick (AD137)
	Ears Action Descriptors (EADs)	Ears forward (EAD101)
		Ears adductor (EAD102)
		Ears flattener (EAD103)
		Ears rotator (EAD104)
		Ears downward (EAD105)
	Eyes and Head Action Descriptors (ADs)	Dogs show the sclera
General behaviours		Tail wagging
		Whine
		Looking away
		Pushing the apparatus
		Sniffing the environment

### Effects on behavioural variables

**Figure 1:**
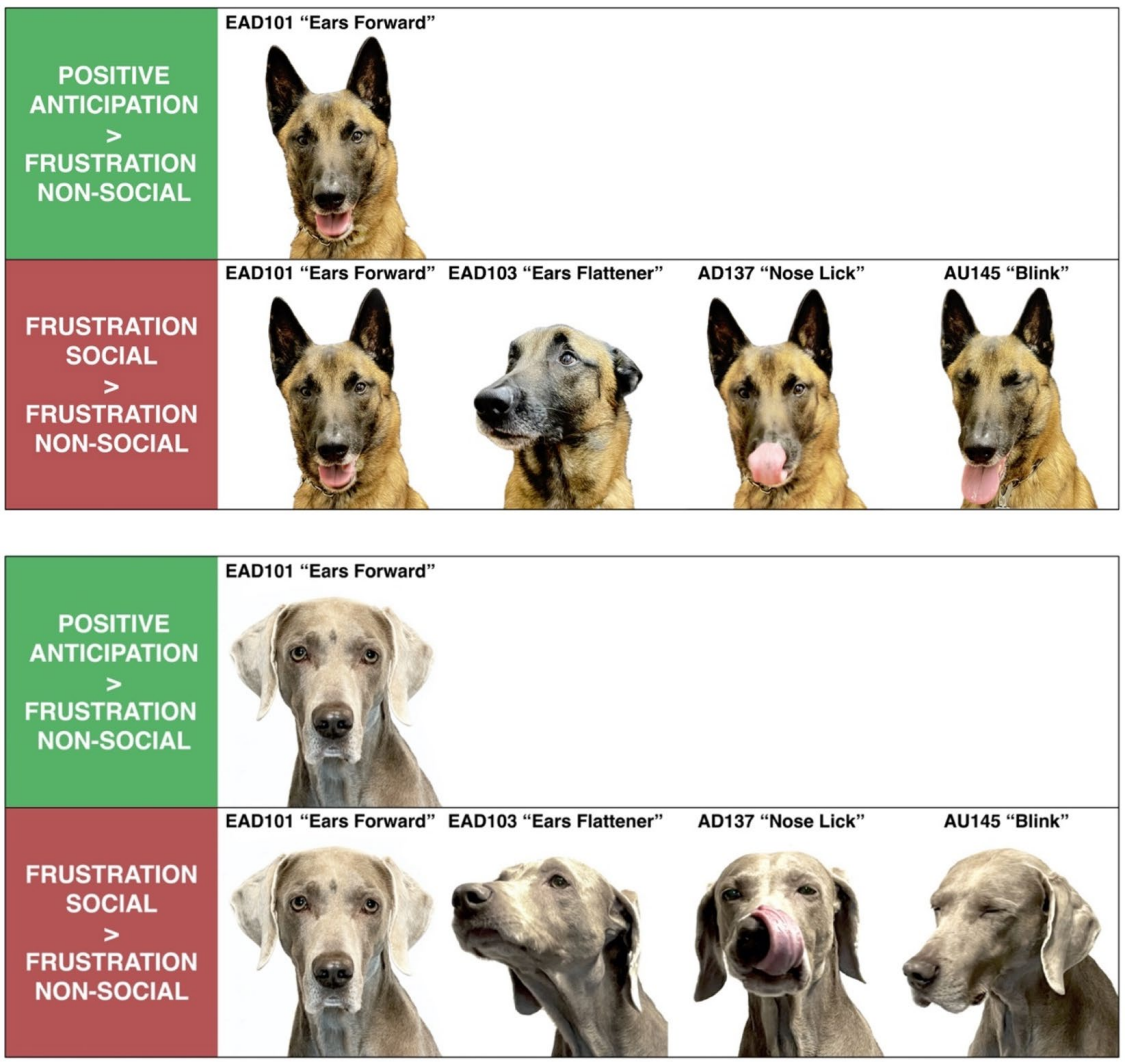
Facial expressions that occurred significantly more in the positive anticipation compared to the frustration non-social (in green) and facial expressions that occurred significantly more in the frustration social compared to the frustration non-social condition (in red). An example of a shepherd type and hunting type dog are depicted.

Test condition had an impact on a number of behavioural variables: “EAD101—ears forward” (full-null model comparison χ^2^ = 17.435, *p* = 0.002) and “pushing the apparatus” (full-null model comparison χ^2^ = 11.418, *p* = 0.022) were more common in the positive anticipation condition compared to the frustration non-social condition (see Table 2 for estimated effects and Fig. 1). “Looking away” (full-null model comparison χ^2^ = 12.631, *p* = 0.002) occurred more in the frustration non-social condition compared to the positive anticipation. “EAD101- ears forward” (full-null model comparison χ^2^ = 17.435, *p* = 0.002), “EAD103—ears flattener” (full-null model comparison χ^2^ = 8.366, *p* = 0.015), “AU145—blink” (full-null model comparison χ^2^ = 22.181, *p* = 0.000), “AD137—nose lick” (full-null model comparison χ^2^ = 8.407, *p* = 0.015), “tail wagging” (full-null model comparison χ^2^ = 12.631, *p* = 0.002) and “whining” (full-null model comparison χ^2^ = 12.552, *p* = 0.002) were influenced by the presence of an audience, occurring significantly more in the frustration social compared to the frustration non-social condition (See Table 2 for estimated effects and Fig. 1). For details about model results see Supplemental Materials Tables from 5 to 19 (page from 10 to 15).

**Table 2:** Main Results of the Binomial regression.

Binomial regression	Positive anticipation compared to frustration non-social			Frustration social compared to frustration non-social		
Behavioural Variables	Estimate	Z	*p* value	Estimate	Z	*p* value
Ears forward (EAD101)	0.822 ± 0.269	3.074	0.002	0.557 ± 0.259	2.153	0.003
Ears adductor (EAD102)	− 0.040 ± 0.199	− 0.199	0.842	− 0.440 ± 0.201	− 2.188	0.029
Ears rotator (EAD104)	− 0.321 ± 0.202	− 1.590	0.112	− 0.257 ± 0.201	− 1.279	0.201
Ears downward (EAD105)	0.370 ± 0.264	1.405	0.160	0.559 ± 0.261	2.142	0.032
Ears flattener (EAD103)	− 0.016 ± 0.542	− 0.003	0.976	1.267 ± 0.478	2.651	0.008
Inner Brow Raiser (AU101)	0.132 ± 0.205	0.642	0.521	− 0.148 ± 0.204	− 0.726	0.468
Blink (AU145)	− 0.380 ± 0.200	− 1.893	0.058	0.492 ± 0.196	2.510	0.012
Showing the sclera	0.106 ± 0.206	0.515	0.607	0.064 ± 0.206	0.309	0.757
Nose lick (AD137)	0.097 ± 0.313	0.310	0.757	0.603 ± 0.289	2.082	0.037
Looking away	− 0.764 ± 0.198	− 3.850	0.000	− 0.147 ± 0.191	− 0.769	0.442
Tail wagging	0.485 ± 0.318	1.525	0.127	2.051 ± 0.334	6.134	0.000
Whining	− 0.679 ± 0.451	− 1.507	0.132	1.020 ± 0.361	2.823	0.005
Sniffing the environment	− 0.762 ± 0.408	− 1.057	0.062	− 0.659 ± 0.397	− 1.057	0.097
Pushing the apparatus	1.566 ± 0.312	5.017	0.000	0.180 ± 0.329	0.546	0.585

The probability of performing “EAD105—ears downward” (Es = − 1.493 ± 0.484, z = − 3.088, *p* = 0.002) and “tail wagging” (Es = − 3.280 ± 1.113, z = − 2.947, *p* = 0.003) was higher in hunting-type dogs compared to shepherd-type dogs while shepherd type dogs were more likely to exhibit “EAD103—ears flattener” than hunting-type dog (Es = 1.850 ± 0.826, z = 2.241, *p* = 0.025). Age affected the occurrence of 3 behavioural variables: “pushing the apparatus” (Es = 0.037 ± 0.113, z = 2.272, *p* = 0.023), “tail wagging” (Es = 0.405 ± 0.197, z = 2.055, *p* = 0.040), “EAD103—ears flattener” (Es = 0.374 ± 0.148, z = 2.530, *p* = 0.011) and “EAD105—ears downward” (Es = 0.219 ± 0.091, z = 2.410, *p* = 0.016) were performed with a higher probability by older dogs compared to younger ones.

The factor “trial” significantly influenced the occurrence of three behavioural variables: “AU145—blink” (full-null model comparison χ^2^ = 15.925, *p* = 0.003), “pushing the apparatus” (full-null model comparison χ^2^ = 11.418, *p* = 0.022), “EAD101—ears forward” (full-null model comparison χ^2^ = 17.435, *p* = 0.002).

### Cortisol and behaviours

For 11 subjects, collected saliva was not enough to measure cortisol concentrations in all conditions, thus these subjects were removed from further analyses. Results of cortisol concentrations measures are hence based on 38 subjects, 17 females (9 non-neutered, 8 neutered) and 21 males (3 sterilized, 18 non-sterilized).

In the positive anticipation test condition 20 dogs had a decrease in cortisol levels during the test while 18 dogs had an increase (mean ∆ = 0.241). In the frustration non-social condition 24 dogs had a decrease in cortisol concentrations during the test while 14 dogs had an increase (mean ∆ = 0.227). Finally, in the frustration social condition, 19 dogs had a decrease in cortisol levels during the test, while 19 dogs had an increase (mean ∆ = − 0.081).

We found no effect of condition on post-test cortisol concentrations (full-null model comparison χ^2^ = 0.960, *p* = 0.619), but in general subjects with higher pre-test cortisol concentrations also had higher post-test cortisol concentrations (Es = 0.302, z = 6.639, *p* = 0.000).

Pre-test cortisol concentrations was associated with the behaviour “looking away” (full-null model comparison χ^2^ = 5.970, *p* = 0.015) and, ear positions “EAD105—ears downward” (full-null model comparison χ^2^ = 10.128, *p* = 0.001) and “EAD101—ears forward” (full-null model comparison χ^2^ = 6.913, *p* = 0.009). In particular, higher pre-test cortisol concentrations were positively associated with the frequency of “looking away” (Es = 0.152 ± 0.060, *p* = 0.012) and the duration of “EAD105—ears downward” (Es = 0.879 ± 0.267, *p* = 0.001) and negatively associated with the duration of “EAD101—ears forward” (Es = − 1.725 ± 0.647, *p* = 0.008). Higher increased post-test cortisol concentrations in dogs were associated with increased environmental sniffing during testing (Es = 0.322, z = 3.238, *p* = 0.001) (for detailed models results see Table 3 and Supplemental Material—tables from 19 to 32 page from 15 to 21).

**Table 3:** Main results of the associations between behavioural variables (frequency and durations) and cortisol concentrations. Results for “sniffing the environment” are related to post-test cortisol concentrations.

Behavioural variables	Positive anticipation compared to frustration non-social			Frustration social compared to frustration non-social			Pre-test cortisol concentration		Post-test cortisol concentration
	Estimate	Z	*p* value	Estimate	Z	*p* value	Estimate	Z	*p* value
Ears forward (EAD101)—duration	2.745	2.303	0.021	2.689	2.243	0.025	− 1.725	− 2.668	0.008
Ears downward (EAD105)—duration	0.035	0.076	0.939	0.227	0.490	0.624	0.879	3.295	0.001
Looking away—frequency	− 0.426	− 2.591	0.010	− 0.077	− 0.506	0.613	0.152	2.510	0.012
Sniffing the environment—duration	− 0.776	− 2.378	0.018	− 0.776	− 2.360	0.018	0.322	3.238	0.001

## Discussion

The main goal of this study was to improve our knowledge of facial expressions and behaviours showed by dogs when tested in situation evoking frustration (a state of negative arousal) or anticipation of a positive event (a state of positive arousal). Specifically, we were interested in understanding whether dogs’ displacement behaviours and facial expressions are linked to a stress response and whether these behaviours and facial expressions are affected by the presence of an audience (in our case an attentive human experimenter).

Following the experimental paradigm by Bremhorst and colleagues^28,29,49^, we tested adult dogs in a situation of anticipation of food delivery and in two situations of frustration during which food was visible but unavailable to dogs and either 1) no human was visible, or 2) a female human was looking at the dog. Furthermore, we collected cortisol saliva samples before and after each test. Several facial expressions and behaviours were affected by the test conditions: ‘ears forward’ and ‘pushing the apparatus’, behaviours associated with attention and interest, occurred more in positive anticipation compared to the non-social frustration condition, conversely “looking away”, a sign of discomfort, was more common in the non-social frustration compared to the positive anticipation conditions. The differences in expression of these behaviours suggest that dogs, as expected, are attentive and interested during the positive anticipation sessions but also that they did not seem to be comfortable during non-social frustration sessions thus turning their head and deflecting their gaze form the apparatus.

Comparing the two frustration conditions, a clear audience effect emerged, with dogs showing significantly more ‘ears forward’, ‘ears flattener’ ‘blink’, ‘nose lick’, ‘whining’ and ‘wagging’ when food was withdrawn with a visible person than without the person being visible. This suggests that these behaviours are not just an expression of frustration (in this case they should have also occurred more in the non-social frustration compared to the positive anticipation one), but rather they could have a communicative function related to the social context.

As for the cortisol measurements, although the post-test concentration of salivary cortisol was not statistically different among the three experimental conditions, thus arguably, the conditions did not elicit a particularly strong stress response in dogs, a number of behaviours were indeed associated with cortisol levels. Dogs with a higher level of pre-test cortisol showed longer duration of ‘looking away’ and ‘ears downward’ and shorter duration of ‘ears forward’, while dogs with higher levels of post-test cortisol showed longer duration of ‘sniffing ground’. These results confirm that a number of behaviours are associated with increased cortisol response. Dogs that started the tests with a higher stress/arousal level (i.e. higher cortisol), potentially showed a reduced attention to the task (shorter duration of ‘ears forward’), and evidenced their slight discomfort with an increased occurrence of ‘looking away’ and ‘ears downward’. These results provide the first direct link between these behaviours and potential physiological indicators of negative arousal.

The association between post-test cortisol and sniffing the environment is also interesting. It may suggest that animals that perceived the test situation as stressful enacted this displacement behaviour, potentially as a coping strategy to entertain or distract themselves during the test.

During the frustration condition Bremhorst and colleagues^28^ found ‘ears flattener’, ‘blink’, and ‘nose lick’ to be more common than in the positive anticipation while we found these patterns to be more common in the social frustration condition when compared to the non-social frustration condition. Taken together these results suggest that ‘ears flattener’, ‘blink’, and ‘nose lick’ are linked to frustration and can be reliable indicator of a negative emotional status but also that they can assume a communicative role when an audience is present.

This is an interesting and novel result since until now only ‘inner brow raiser’ (also known as ‘puppy dog eyes’), ‘tongue show’, and ‘vocalization’ were reported to be performed by dogs more when a person was attentive (looking at the dog) compared to when she was turning her back to the dog^48^. Kaminski and colleagues interpreted these behaviours and facial expression as an active attempt to communicate with social partners rather than involuntary displays of emotional states. However, a subsequent study by Bremhorst and colleagues^49^, comparing dogs’ facial expressions during a social and a non-social food delivery task, demonstrated that ‘inner brow raiser’ is simply correlated with the eye movement, giving a lower-level explanation for its higher occurrence in social contexts. We did not find any significant effect of test condition on the occurrence of this pattern further casting doubts on the communicative intent of this behaviour. Unfortunately, Bremhorst and colleagues in their study^49^ considered only this facial expression thus it is not possible to compare other behavioural patterns emerged in our study as characteristic of a social frustration condition. Ears flattener was previously associated with fear^27^ but also identified as an appeasement signal or an agonistic response to a perceived threat in dogs^20^ and as an active and passive submission signal in wolves’ intraspecific interactions^50^. The position of the ears, combined with other body postures has been identified as a principal component of canid visual communication and emotional expressions^51^, however experimental testing of whether they are indeed more likely to occur in social contexts have been lacking. Given our results it is possible that these facial displays are exhibited as attempts to interact with the experimenter. However, given the communicative valence of these signals, our experimental paradigm cannot disentangle the dogs’ underlying motivation (appeasement, submissive, requesting) which may have elicited these behaviours, thus further investigations will be needed in future.

Two displacement behaviours were also observed to occur more often in social vs. non-social context i.e., “nose lick” and “blink”. The behaviour of “nose lick” was previously identified as an appeasement signal^43^ in dogs and as a friendly/submissive signal associated with greeting conspecifics in wolves^50^. Furthermore, it is commonly identified as a stress indicator, and found to be positively correlated with salivary cortisol in dogs during an hospital stay^52^, but negatively correlated with urinary cortisol/creatinine ratio and associated with de-arousal in boarding kennels^53^. We did not find any correlation of pre-test or post-test cortisol concentrations with the performance of nose lick, suggesting that the only factor eliciting the behaviour was the presence of the social partner. Further investigation, controlling for humans’ or other dogs’ presence, are needed to clarify the arousal influence and to confirm the communicative function of this self-directed displacement behaviour. Furthermore, a clear distinction between the behaviours of nose lick and lip wipe as the one adopted by the DogFACS manual and the present study, is recommended in future studies, allowing a clear recognition and a possible distinction in their nature and function.

Blinking has been previously associated with fear^27^ and frustration^28^ in dogs. In our studies we didn’t find a significantly higher occurrence of blink in the frustration non-social condition compared to the positive anticipation, but we found it to be significantly influenced by the visibility of the experimenter. The experimenter looked directly at the dog during the test, although they did not adopt a threatening gaze or posture, it is possible that the direct gaze may have been perceived by dogs as a mildly threatening behaviour and that they therefore interrupted the eye contact closing the eyes and thus blinking more in the social condition. However, cortisol levels did not differ between conditions, suggesting that the social condition was not strongly associated with a strong increase in negative arousal, thus this interpretation will require confirmation in future studies.

Differences between the present and the original experimental protocol by Bremhost and colleagues^28^ make it difficult to compare results that appear to be somehow different: we added a new condition, (i.e. social frustration), reduced the duration of the frustration sessions (from 60 to 25 s) and tested dogs with two distinct ear morphology (erected vs floppy ears). A reliable indicator of positive anticipation was ‘ears adductor’ in their original study (and the one by Caeiro and colleagues^30^) while we found ‘ears forward’ to be more common during the positive anticipation; although different, both these ears’ positions could be indicative of the attention given by dogs to the apparatus/task. The likelihood to show ‘ears adductor’ and ‘ears forward’ are not different between dogs with erected or floppy ears, but it is possible that the focus on one breed, Labrador Retrievers tested by Bremhost and colleagues^28^, may have highlighted breed-specific characteristics in the use of facial expression which may not be generalizable to other dog breeds/morphological types. On the other hand, ears forward have previously been associated with general attention, alertness, and confidence^9,20^ and thus not necessarily associated with the positive emotional valence of the test. The fact that, in our study, ears forward was more common in the positive anticipation and in the frustration social situation (when a human looking at them was visible to the dog) and that this position was negatively associated with the pre-test cortisol concentration supports the hypotheses that more confident dogs were more focused during the positive anticipation and social frustration test.

Other displacement behaviours such as scratching, stretching, auto-grooming and yawning (stress related behaviours^31,33,40,54^) occurred too rarely during the test to allow for statistical analyses. This may be due to a low degree of frustration caused by the denial of food; longer frustration sessions may allow higher occurrence of displacement behaviours. Finally, the general behaviours of tail “wagging” (performed more by hunting-type dogs) and “whining” were also more likely to occur with an audience. Both behaviours were previously found to be influenced, even if not to a statistically significant degree, by the attentiveness of a human experimenter^48^. Different kinds of tail wagging (tail carried high or low, wagging fast or slow) have been considered as visual communicative signal used to emphasise signals expressed by facial and body postures, or with vocalization^20^. Tail wagging has been associated with arousal^33,35^ and the expectation of positive stimuli^55,56^. Furthermore, lateralized tail wagging has been associated with different emotional correlates^57,58^. Our results highlight the strong influence of an audience on the performance of tail wagging, while there were no differences between the positive anticipation and the frustration non-social condition and no influence of cortisol concentrations, suggesting no direct correlation with arousal levels.

Based on the current study, it is not possible to disentangle whether these putative communicative signals (expressed in the presence of a human) are a product of domestication, the result of the intense socialization and exposure of pet dogs to humans or a combination of above. In fact, the expression of behavioural displays could be the output of a learned discrimination process in which some signals (e.g., tail wagging) are consciously or unconsciously rewarded by humans. Further studies considering similarly raised wolves and dogs and dog populations with differing exposure to humans may help to answer such questions.

Another limitation of our study is the absence of a positive anticipation social condition. On one hand, the presence of a positive anticipation social condition would have completed the design of the experiment, trying to disentangle positive and negative putative communicative signals. On the other hand, even if the order of the test session had been counterbalanced, the association between the presence of the human and the reward or between the presence of the human and the denial of the reward could have influenced the subject’s subsequent test session. Thus, we would not have been able to exclude a biased emotional state in the second session with the human present. Given the gaps in the literature that identified the same signals as negative valanced stress signals and appeasement behaviours (communicative signals performed in a potential conflictual situation), the aim of the study was to test in a clear and standard manner the specificity of signals and expressions to a negative valence arousal situation (compared to a positive valanced arousal situation) and to the presence of a potential social partner only in a negative valanced situation, for which we had clear predictions. Nevertheless, future studies can adopt this methodology to address these questions.

In conclusion, we show that positive and negative arousal evoking situations and the presence of an audience affects dogs’ behavioural displays and facial expressions in different ways. The fact that many behavioural and facial displays were elicited by the presence of an attentive human experimenter suggests that dogs are sensitive to the social context, and they can flexibly exhibit specific displays. We should then be aware that the classification of these behaviours as mere stress-related signals could be misleading regarding dogs’ real motivations and intentions. Nevertheless, their classification as “appeasement signals” is also premature since few studies specifically tested the different social context in which they occur (e.g. conflict driven vs. no conflict) and the feedback they elicit in the receiver. Furthermore, the degree to which the dog is in control of its signalling and its dependence on different audience characteristics and feedback will require further investigation. Humans may be considered a source of stimulation, as well as an audience, hence behavioural displays may vary in relation to the type of audience present (e.g. conspecific, heterospecific) and the associations with such stimuli. Thus, further studies should always consider the component of arousal, emotional valence and presence of potential social partners when investigating dogs’ behavioural responses to different potentially stressful stimuli. The aim of future research should be to clarify the function of these display, whether they are sensitive to intra and interspecific contexts (their dependence on different audience characteristics) and the feedbacks they elicit in their potential receiver (especially conspecifics).

## Materials and methods

### Ethical statement

All the procedures were approved by the Animal Welfare committee of the University of Parma in accordance with Animal Welfare organization (OPBA) and ARRIVE guidelines and regulations (Protocol number PROT. N. 6/CESA /2022). Informed consent for experimental procedure and for publishing images and findings in an open access publication was obtained from the owners.

### Subjects

We tested 49 subjects, 24 females (14 neutered and 10 intact) and 25 males (4 neutered and 21 intact) (mean age:5.3, min:1, max:10). Two types of dogs, based on morphological and phylogenetic criteria, were recruited. Shepherd-type dogs with erect ears, long and triangled snout and hunting-type dogs with floppy ears, soft skin around the mouth region and a squared face. We tested 24 hunting-type dogs and 25 shepherd-type dogs. Behavioural analyses were performed on 49 subjects, while analysis including hormonal correlates were conducted on 40 subjects since not enough saliva for cortisol analyses was collected for 9 dogs (see Table 1—Supplemental material). For purpose of saliva collection and measurements of cortisol levels, owners were instructed avoid exercising and feeding the dog for 2 h before the test and avoid them drinking 15 min before the test.

### Experimental design and set up

**Figure 2:**
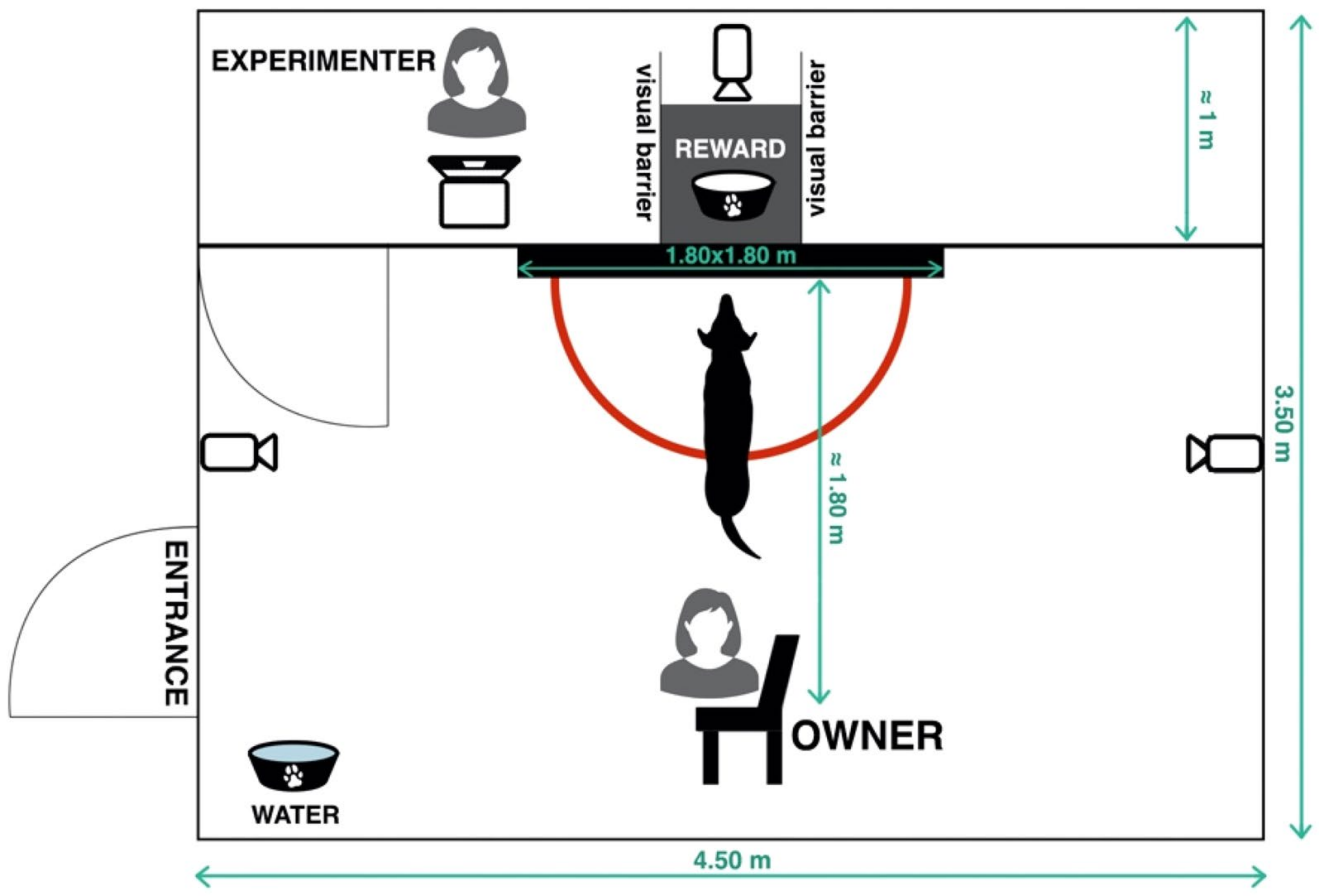
Set up of the room used for the tests.

The experiment was conducted in an indoor room (4 × 7 m) at the Dog’s Ethology Lab of the University of Parma, between February 2021 and July 2021. We adopted a within subject design, in which each dog was tested 3 times on different days, approximately at the same time of day to control for cortisol circadian rhythm fluctuation^59^. The room was divided into two parts by a wooden and plastic apparatus (Fig. 2). The central part of the apparatus was a 1.80 × 1.50 cm panel with a 50 × 100 cm open window in the middle at 30 cm height from the ground. On the two sides of the window two wooden bars were fixed to keep the dog in the focus of the camera during the test. The window was covered by two movable panels: a Plexiglas one and an opaque one. A table measuring 30 cm in height and 50 × 100 cm wide was placed directly behind the window. On the sides of the table two white panels covered the rest of the room from the sight of the dog. On the table, a stick with a plate attached at the end could be moved by an experimenter placed on the right side of the table, behind the white panel (See Fig. 2). Behind the apparatus, on the right side of the panel a computer connected to 3 different cameras was installed to allow the experimenter to see the dog and the owner without being seen by them. In front and at 3 m distance from the panel, a chair was placed for the owners. Water was available for the dogs during the test procedure but not during the post-test waiting period (15 min). Three cameras allowed the experimenter to monitor the dog: one of them was settled behind the apparatus and the table, focused on the dog face, the remaining two were placed on the right and the left side of the wall, to record every dog’s activity.

### Experimental procedure

Every test session lasted about 30 min. On arrival in the testing room, the owner was asked to un-leash the dog, allowing it to roam free, explore the environment, and familiarize with the experimental set-up. Having compiled the research consent form, the owner was asked to take a seat wearing a facial mask and sunglasses and to ignore the dog for the duration of the test sessions. Before starting the test one of the two experimenters collected the first saliva sample and stored it in the freezer (at − 20 °C). Then, the experimenter went behind the apparatus, checked the computer, and prepared ten food slices for the habituation phase.

The first part of each session was the habituation phase, intended to put all dogs in the same motivational state before each test condition. After the habituation phase, one of the three test conditions was performed. The dogs experienced different test conditions in a counterbalanced order, meaning that each subject experienced the tests condition in different orders (6 possible combinations), and different conditions were performed within the same test day, depending on each dogs’ order. The three test sessions were performed at the same time on different days, within 2 weeks depending on the availability of the owners, a part for 4 dogs which had to wait 55–57 days for the last test condition due to COVID lockdown measures.

In the habituation phase, after having placed the food in the plate on the table, the opaque panel was opened by the experimenter, allowing the dog to see but not reach the food for 3 s. After that, the plexiglass panel was opened, and the dog was allowed to get the food reward. This procedure was repeated for 10 consecutive trials. If the dog retrieved the food for the 10 habituation trials the test session started. If the dog didn’t show interest in retrieving the food reward or was too afraid of the apparatus to stay close to it, it was excluded from the study (this occurred for 4 dogs).

The tests consisted of five identical consecutive trials per condition, each trial lasting 5 s.

**Figure 3:**
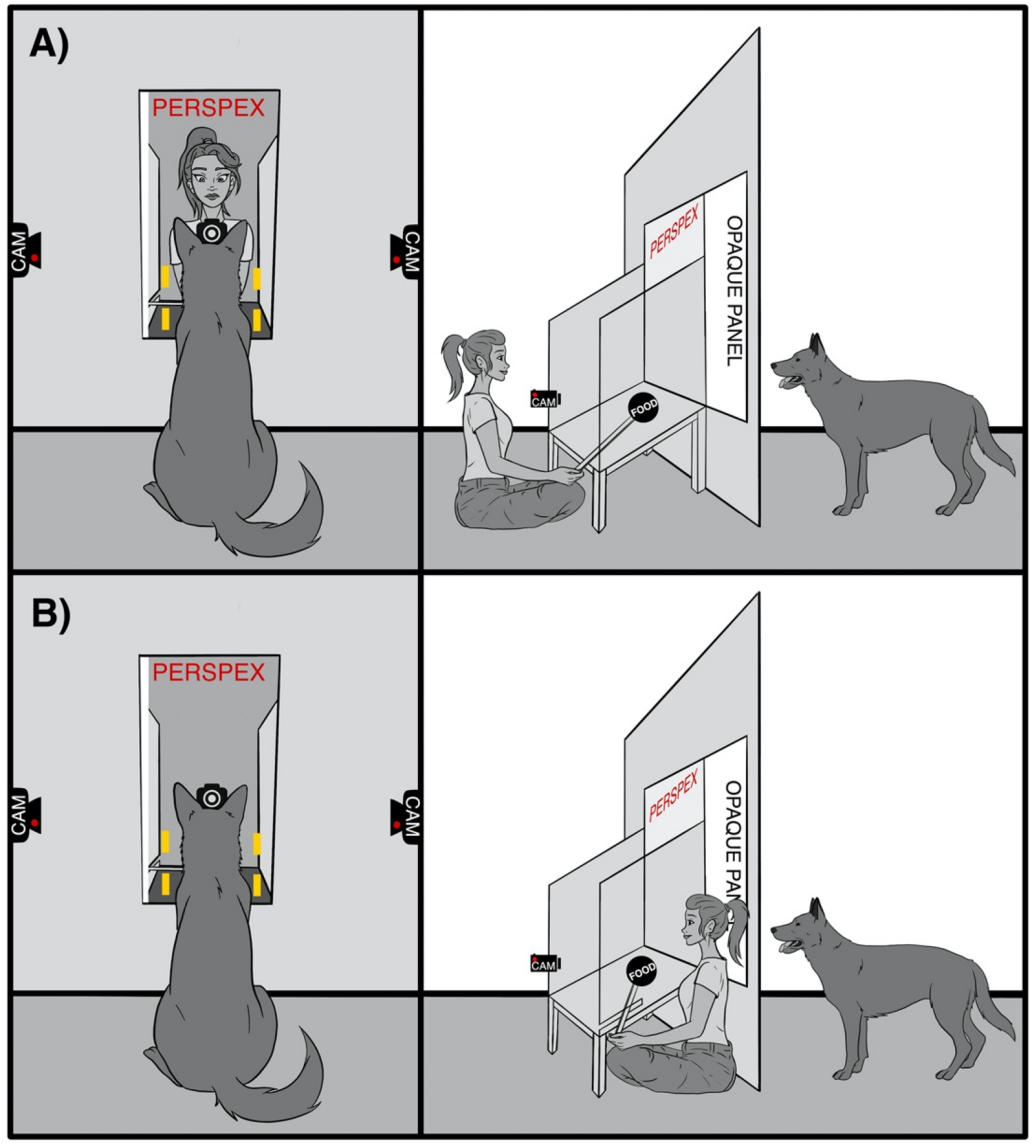
Experimental apparatus, (**A**) experimenter position during the frustration social condition, (**B**) experimenter position during the frustration non-social and positive anticipation condition.

- **Positive anticipation test (PA)**: While the opaque panel was closed the food reward was placed in the movable plate on the table by the non-visible experimenter. After that, she opened the opaque panel allowing the dog to see but not reach the food reward for 5 s. The transparent panel was then opened, and the dog was able to retrieve the food. As soon as the dog finished eating, both panels were cautiously closed, and the plate was refilled with another piece of food for the next trial. The experimenter was never visible.
- **Non-social frustration test (FN)**: The negative high arousal situation was provoked by five trials of food denial. Once the food reward was put inside the plate behind the openable window at the centre of the apparatus, the experimenter opened the opaque panel and let the tested dog see the bowl with the food reward. After 5 s the experimenter moved the plate with the reward away from the dog, the plate was visible to the dog but not reachable for 5 s. At the end of this period the opaque panel (the only one ever opened) was closed and another trial started. The plexiglass panel remained closed for the whole FN test phase, preventing the dog from reaching the food. The experimenter was never visible.
- **Social frustration Test (FS)**: The high arousal negative social situation was provoked by five trials of food denial by a visible experimenter staring at the dog (See Fig. 3). Once the food reward was put inside the plate, the experimenter positioned herself behind the table and opened the opaque panel. The experimenter was in full view of the dogs, positioned behind the frontal camera and she remained still while staring at the dog. After the first 5 s in which the food was visible and near the dog, the experimenter moved the food away from the dog and towards herself. Then, she stayed still looking at the dog for more 5 s (the social frustration phase). At the end of this period the experimenter closed the opaque panel and the next trial started. The Plexiglas panel remained closed for the whole FS test phase, preventing the dog from reaching the food. The experimenter was always **visible** during the 5 trials.

At the end of each test phase the owner was asked to stay with their dog for another 15 min in the experimental room: during this time no water nor food were provided to the dog tested. After 15 min the second (post-test) saliva sample was collected (time needed for cortisol levels to rise after the stressful event^37^). After the last saliva sample collection, the dogs underwent two positive trials in which it could get the food reward to leave the laboratory with a positive experience.

### Hormonal measures and laboratory analysis (cortisol analysis)

Saliva samples were taken using Salivettes (Sarstedt, Ges.mbH, Wr. Neudorf, Austria) to measure cortisol concentration (i.e., a validated physiological indicator of stress in dogs^34,60^). The collecting method foresaw the insertion of the swab inside the dogs’ mouths for a few seconds, until it was saturated. To induce salivation, a slice of sausages was presented to the dog, allowing them to sniff (but not eat) it just before using the swab. Pre-test and post-test saliva samples were collected for each subject in each test condition. All samples were named, numbered, dated, categorized as “pre” or “post” test and stored at − 20 °C until the analysis. They were then thawed at room temperature for 30 min and centrifuged for 15 min at 20 °C at 1500 rpm. A cortisol enzyme immunoassay^61^ was used to analyse cortisol levels. Each plate of the assay contained all the samples of 6 subjects and we used 8 plates. This assay has been validated and successfully utilized in previous studies^62,63^.

### Behavioural coding

Dogs’ behavioural displays and facial expressions were video recorded by three cameras synchronized with OBS software (https://obsproject.com). The videos were then coded using Solomon Coder Beta 15.01.2013 (Andrá Péter, http://solomoncoder.com).

Dogs’ facial expressions were coded with DogFACS (Facial Action Coding System) an anatomically based system for the scientific measurement of facial movements^28,30,64,65^. DogFACS behavioural variables included Action Units (AUs), actions including the use of a single muscle or a group of muscles, such as movements of the upper and lower face (e.g. blink, inner brow raiser, mouth movements) and Action Descriptors (ADs), movements usually requiring the use of several muscles or that involve actions, like ears and head movements (see Table 2 and 3—Supplemental material—for the detailed ethogram). Even though based on the DogFACS manual, dogs with floppy ears should not perform “Ears Rotation”, in our study, this movement was observed both for shepherd-type dogs with pointy ears and for hunting-type dogs with floppy ears (See Figure 1—Supplemental Materials); thus, it was coded for both. A second ethogram including postures, locomotory activities, vocalization, and displacement behaviours (self-directed and environment directed) was used to code general behaviours (See Table 4—Supplemental material- for detailed ethogram). Videos were coded by two certified DogFACS experimenters (GP and CC). Both coders analysed 72 videos (20% of the videos) to assess inter-rater reliability. Intra-class correlations (ICCs;^66^) were performed in R 4.1.0 (function: “ICC”; package: psych). Ears Action Descriptors, Displacement behaviours and Vocalization had a moderate strength of inter-rater agreement (EADs and Events: 0.70 < ICC < 0.75), all the other variables showed an excellent reliability (0.85 < ICC < 0.95).

### Statistical analysis

DogFACS units and behaviours observed in at least 10% of dogs in at least one of the three test conditions were used for statistical analyses (See Table 1). Facial movements related to the opening of the mouth AU116—Lower lip depressor, AU25—lips part, AU26—jaw drop and AD126—panting, were excluded from further statistical analyses because they can be influenced by temperature and dogs were tested in different seasons.

To investigate which behaviours and facial expressions were specific to a frustration evoking situation, compared to a positive anticipation situation, and which were dependent on the presence of an audience, we computed generalized linear mixed models (GLMMs^67^) with binomial distribution using the function “glmmTMB” of the package “glmmTMB”. The likelihood of occurrence of a behaviour in each of the 5 trials of the test sessions was used as a response variable to investigate the specificity of behaviours to the different test conditions: whether the positive/negative/negative-social stimuli would have elicited (yes/no) a particular behaviour, thus being specific of that context. Thus, the unit of occurrence were the 5 s of each trial, and the trial number influence was assessed for each behavioural variable testing the significance of the factor “trial” with a full-null model comparison (see Tables 5-18 Supplemental Materials). The test condition (3 levels variable: positive anticipation, frustration non-social, frustration social) was entered as the test predictor variable, and several variables (dog type (hunting or shepherd), age and sex) were included as fixed control effects. Different facial morphology related to dog type and phylogenesis (es. hunting or shepherd dog breeds) may affect the manifestation of specific facial expression and their detection by observers^65,68^. Furthermore, all previous studies using DogFACS statistically or experimentally controlled for dogs’ facial morphological features (testing only dogs of one breed^29^; including ears morphology as the control in statistical analysis^30^). Therefore, dog type (hunting-type/shepherd-type) was included as control predictor. Older dogs have been shown to be more anxious and avoidant of some novel situations^69^ and sex differences in personality traits, inclination to social interaction with humans and visual focus have been found in domestic dogs^70^. Since these factors could influence behaviours in our test conditions, we included age and sex as control predictors in all the models.

The subject ID was included as random intercept effects to control for repeated sampling. A likelihood ratio test comparing the full model with a null model lacking the main test predictor “condition” was performed to keep the type I error rate at 5%.

To compare the three different levels of the test predictor “condition” (levels: frustration non-social, positive anticipation, frustration social) the level “frustration non-social” of the test predictor “condition” was set as the reference level.

To assess the influence of different trials (we had 5 identical trials for each test condition) on the occurrence of behavioural variables we performed a likelihood ratio test comparing the full model (comprising only “condition” as test predictor) with a model including also the factor “trial” as test predictor.

To rule out collinearity, Variance Inflation Factor (VIF^71^) was determined with the function “vif” of the package car (version 3.0-0), applied to all models lacking the random effects. It revealed no higher values than 1.2. We checked overdispersion with the function "check_overdispersion”. Models were not overdispersed (overdispersion ratio < 1 for all the models). We assessed model stability on the level of the estimated coefficients and standard deviations by excluding the levels of the random effects one at a time^72^. This revealed the models to be of good stability (See Supplemental Materials for detailed results). Parametric bootstrapping was performed to obtain confidence intervals (function “boot.glmmTMB”).

A preliminary investigation regarding the influence of the test-conditions (positive anticipation, frustration non-social and frustration social) on post-test saliva cortisol concentration was conducted computing a GLMM with Gaussian error structure using the function “glmmTMB” of the package “glmmTMB”. We included post-test cortisol (log-transformed) as the response variable and the three levels of test conditions as test predictor. Pre-test cortisol concentration, age, sex and neutered status of the subject were included as control predictors. To assess the general influence of different test conditions on post-test cortisol concentration, using a likelihood ratio test^73^, full model was compared with a null model^74^ lacking the test predictor “test conditions” but comprising the control predictors and random effects (subject ID)^74^. Collinearity was assessed using the function “vif” of the package car (version 3.0-0), applied to the model lacking the random effects. It revealed no higher values than 1.12.

Since post-test saliva cortisol concentrations were not influenced by the test conditions, but only by pre-test saliva cortisol concentrations (see section *Results*), we investigated whether pre-test saliva cortisol concentrations would be associated with higher performance of behavioral variables. The sample for these models encompassed 282 cortisol values, taken from 44 individuals across the 3 days of testing. We ran generalized linear mixed models with the function “glmmTMB” of the package “glmmTMB” with a Gaussian distribution error structure for duration variables and with a Poisson distribution error structure for frequency variables. The sum of the durations or the frequencies of the behaviours in all the 5 trials was modelled as a function of the pre-test cortisol concentration (test predictor). Post-test cortisol concentration, test condition, age, sex and dog type were included as control predictors. The subject ID and plate ID were included as random factors in all the models since we had repeated observation of the same individual and we analysed cortisol samples in 8 plates containing 6 subjects each.

To assess the general influence of pre-test saliva cortisol concentrations on the expression of behavioural variables, full models were compared with null models^74^ lacking the test predictor pre-test cortisol concentrations but comprising the control predictors and random effects using a likelihood ratio test^73^. Collinearity was assessed using the function “vif” of the package car (version 3.0–0), applied to the model lacking the random effects. It revealed no higher values than 1.64. For Poisson models overdispersion was checked with the function "check_overdispersion”. It revealed models to not be overdispersed (overdispersion ration < 1; only the model for AD137—lips licking has an overdispersion ration: 1.2). Model stability was assessed on the level of the estimated coefficients and standard deviations by excluding the levels of the random effects one at a time^72^. This revealed the models to be of good stability (See Supplemental Materials for detailed results). Parametric bootstrapping was performed to obtain confidence intervals (function “boot.glmmTMB”). All statistical analyses were performed in R (version 3.6.1; R Core Team 2019). Results were considered statistically significant if *p* ≤ 0.05.

## Supplementary Information

Supplementary Information.

## Data Availability

The raw data and analysis of this study are available from the corresponding author on request.
