# Supplementary material for: Audience effect on domestic dogs’ behavioural displays and facial expressions
Source: Sci Rep. 2022 Jun 13;12:9747. doi: 10.1038/s41598-022-13566-7 (PMC9192729; doi:10.1038/s41598-022-13566-7)
Supplement: Supplementary file 1 — Supplementary Information. [file 41598_2022_13566_MOESM1_ESM.docx]

**Supplemental Material**

Table 1 – Subjects

|  | **subject** | **age** | **sex** | **castration** | **Breed/dog type** | **Session order** |
| --- | --- | --- | --- | --- | --- | --- |
| 1 | Aaron | 1.5 | m | No | Rhodesian Ridgeback | positive/frustration_social/frustration_nonsocial |
| 2 | Alaska | 2 | m | No | Pastore Lupino  del gigante | frustration_nonsocial/positive/frustration_social |
| 3 | Alì | 6 | m | Yes | Kurzhaar | frustration_social/positive/frustration_nonsocial |
| 4 | Alma | 2 | f | No | Border collie | frustration_social/positive/frustration_nonsocial |
| 5 | Amelia | 3 | f | No | Weimaraner | frustration_nonsocial/frustration_social/positive |
| 6 | Ares | 9 | m | No | Border collie | frustration_social/positive/frustration_nonsocial |
| 7 | Argo | 4 | m | No | Australian Shepherd | frustration_social/frustration_nonsocial/positive |
| 8 | Artù | 8 | m | No | Cocker Spaniel | frustration_nonsocial/positive/frustration_social |
| 9 | Belen | 4 | f | Yes | German Shepherd | frustration_nonsocial/positive/frustration_social |
| 10 | Bella | 3 | f | Yes | Kurzhaar | frustration_social/frustration_nonsocial/positive |
| 11 | Benito | 3 | m | No | German Shepherd | frustration_social/positive/frustration_nonsocial |
| 12 | Boris | 2 | m | No | Australian Shepherd | frustration_social/frustration_nonsocial/positive |
| 13 | Bughi | 4 | m | No | Kurzhaar | positive/frustration_social/frustration_nonsocial |
| 14 | Chico | 6 | m | No | German Sheperd | frustration_nonsocial/positive/frustration_social |
| 15 | Ciro | 4 | m | No | Labrador | frustration_nonsocial/frustration_social/positive |
| 16 | Demon | 7 | m | No | Weimaraner | frustration_nonsocial/frustration_social/positive |
| 17 | Dusty | 6 | f | Yes | Border collie | frustration_social/positive/frustration_nonsocial |
| 18 | Dylan | 5 | m | No | Shetland Sheperd | positive/frustration_nonsocial/frustration_social |
| 19 | Eve | 7 | f | Yes | Labrador | frustration_social/frustration_nonsocial/positive |
| 20 | Gemma | 7 | f | No | German Sheperd | frustration_nonsocial/frustration_social/positive |
| 21 | Gigia | 1 | f | No | Labrador | frustration_nonsocial/frustration_social/positive |
| 22 | Hope | 4 | f | Yes | Vizsla | frustration_social/positive/frustration_nonsocial |
| 23 | Indi | 10 | f | Yes | Border collie | positive/frustration_social/frustration_nonsocial |
| 24 | Iris | 9 | f | Yes | Labrador | frustration_social/frustration_nonsocial/positive |
| 25 | Jago | 7 | m | No | Border collie | positive/frustration_social/frustration_nonsocial |
| 26 | Junior | 2 | m | No | Labrador | frustration_social/frustration_nonsocial/positive |
| 27 | Kuma | 10 | f | Yes | Pastore belga | frustration_social/positive/frustration_nonsocial |
| 28 | Lady | 5 | f | Yes | English Setter | positive/frustration_nonsocial/frustration_social |
| 29 | Leone | 2 | m | No | Weimaraner | frustration_social/frustration_nonsocial/positive |
| 30 | Loba | 6 | f | No | German Sheperd | positive/frustration_social/frustration_nonsocial |
| 31 | Lola | 4 | f | Yes | German Sheperd | positive/frustration_social/frustration_nonsocial |
| 32 | Malva | 8 | f | Yes | Border collie | frustration_social/frustration_nonsocial/positive |
| 33 | Marta | 9 | f | Yes | mix breton/setter | frustration_nonsocial/frustration_social/positive |
| 34 | Milla | 1 | f | No | Lupino del Gigante | positive/frustration_social/frustration_nonsocial |
| 35 | Nervo | 6 | m | No | Malinois | frustration_nonsocial/frustration_social/positive |
| 36 | Newton | 4 | m | Yes | Toller Retriver | frustration_nonsocial/positive/frustration_social |
| 37 | Olimpia | 3 | f | Yes | Rhodesian Ridgeback | frustration_nonsocial/positive/frustration_social |
| 38 | Olivia | 1 | f | No | Rhodesian Ridgeback | positive/frustration_nonsocial/frustration_social |
| 39 | One | 9 | m | Yes | Weimaraner | frustration_social/positive/frustration_nonsocial |
| 40 | Paco | 7.5 | m | Yes | Weimaraner | frustration_nonsocial/positive/frustration_social |
| 41 | Paco1 | 9.5 | m | No | Border collie | frustration_nonsocial/positive/frustration_social |
| 42 | Quickly | 7 | m | No | Border collie | frustration_nonsocial/frustration_social/positive |
| 43 | Ringo | 8 | m | No | Belgian Sheperd | frustration_social/positive/frustration_nonsocial |
| 44 | Rumba | 5 | f | Yes | Kurzhaar/ Labrador mis | positive/frustration_nonsocial/frustration_social |
| 45 | Samuel | 5 | m | No | Pointer | positive/frustration_nonsocial/frustration_social |
| 46 | Tilde | 4 | f | No | Pastore del Lagorai | positive/frustration_social/frustration_nonsocial |
| 47 | Uma | 3 | f | No | Rhodesian Ridgeback | positive/frustration_nonsocial/frustration_social |
| 48 | Whisky | 10 | m | No | Australian Shepherd | frustration_nonsocial/positive/frustration_social |
| 49 | Ziva | 6.5 | f | No | Weimaraner | positive/frustration_social/frustration_nonsocial |

**Table 2** – DogFACS Ethogram part.1

| **General Category** | **Category (mutually exclusive behaviours)** | **Code** | **Definition** | **Description** | **Type of measure** |
| --- | --- | --- | --- | --- | --- |
| ACTION UNITS (Facial movements) | ACTION UNITS  (AU) - Upper Face | AU101  (R-L) | Inner Brow Raiser | When the inner brow raises it can be distinguished a dorsal movement of the protuberance on the inner eye. The dog raises the soft protuberance present above the inner corner of the eye. Eyes become rounder and wider, and a vertical wrinkle may appear just between them. The left and the right region of the brow can be moved separately (**AU101-R** or **AU101-L**) or together (**AU101**). | Duration |
|  |  | AU143 | Eye Closure | Upper and lower eyelids move towards together until they completely touch each other when the eyes close completely. Eyes remain closed for **at least half a second**. | Duration |
|  |  | AU145  (R-L) | Blink | Upper and lower eyelids move towards together until they completely touch each other when the eyes close completely. In the AU145 **eyes open again within half a second**. The left and right eye can be closed indipendently (lateralized movement) (AU145-R, AU145-L) or together (AU145). | Event |
|  | ACTION UNITS (AU) - Lips Lower Face I | AU109+110 | Nose wrinkle and Upper lip raiser | In dogs the nose wrinkle (**AU109**) is only observed with AU110 (upper lip raiser) since they have only three muscles (levator nasolabialis, caninus, levator labii maxillaris) that work together for raising the upper lip and pulling the nose dorsally and/or caudally. These movements usually induce wrinkles formation on the muzzle and often come together with a snarl, causing the display of upper teeth. The upper lip raiser (**AU110)** can also be seen without the nose wrinkle (AU109), hence to the raise of the levator nasolabialis muscle alone. It can be observed with AU26 or AU27. | Duration |
|  |  | AU110 | Upper lip raiser |  | Duration |
|  |  | AU12 | Lip corner pull | Due to the contraction of the zygomaticus muscle the lips' corners are pulled towards the ears, curving slightly.  The mouth is opened, more teeth are visible, and some wrinkles may appear around the lips' corners as they're stretched. | Duration |
|  |  | AU118 | Lip pucker | The dog pushes the corners of its mouth forward (rostrally), the muzzle stays tense and some theet may be visible if the mouths slightly opened. | Duration |
|  | ACTION UNITS (AU) -Lips Lower Face II | AU116 | Lower lip depressor | Contrary to the neutral state with mouth open, in whom only the tips of the canines are visible, this movement makes other lower teeth, sometimes even the gums, visible as the lower lip's withdrawn ventrally. | Duration |
|  | ACTION UNITS (AU) - Lower Face III | AU26 | Jaw Drop | The lower jaw is dropped without any sign of tension (mouth sglightly open).  **AU26** can been coded even without a clear sign of lips separation, but teeth separation must be seen or at least perceived. | Duration |
|  |  | AU27 | Yawning (Mouth Stretch according to DogFACS) | The mouth is stretch opened, and the lower jaw tensed (mutually exclusive of the AU26). Teeth are exposed, the tongue and the oral cavity are shown, and the lips are pulled back. | Event |
|  |  | OPEN_CLOSE | Gasping | The dog opens and closes its mouth repetitively in a very short amount of time (at least three times within max 2 seconds). | Duration |

**Table 3** – DogFACS Ethogram part.2

| ACTION DESCRIPTORS (AD) - Mouth | AD19 | Tongue Show | The dog shows its tongue, the mouth is opened with the lower jaw dropped and lips parted. | Duration |
| --- | --- | --- | --- | --- |
|  | AD37  (L or R) | Lip wipe  (Lips licking)  (lateralized movement) | The tongue wipes the lips from the mouth midpoint till the mouth corner. This movement is usually lateralized (**AD37-R** or **AD37-L**). If the tongue is licking the lips just at the midpoint code as **AD37**. | Event |
|  | AD137 | Nose lick | The jaw is lowered, and the tongue licks the nose. It can be followed by a AD37 but they should be coded separately. Also flicks of the tongue are included. | Event |
|  | AD126 | Panting | Tongue's shown, the lower jaw is dropped and there is no sign of tension.  The dog breathes quickly and noisily through its mouth, while its chest moves rapidly. | Duration |
|  |  |  |  |  |
|  |  |  |  |  |
|  | AD33 | Blow | Cheeks swell briefly just before the air's blown out, then the dog gets back to a neutral expression. | Event |
|  | AD35 | Suck | The dog holds in its mouth, or sucks against the teeth, a piece of its own cheek. Slightly visible, it can be coded alone or with the AU26. | Event |
| ACTION DESCRIPTORS (AD) - Eyes | SCLERA | The dog's showing the sclera | As the dog changes the direction of its gaze the sclera may turn visible, which make easier to understand the movement of the eye. The visibility of the sclera and direction of the gaze is an important social cue that helps dogs to understand conspecifics and humans appropriately. | Duration |
| ACTION DESCRIPTORS (AD) - Head | AD55 | Head tilt left | The dog's focused and then head is tilted to the left side. | Duration |
|  | AD56 | Head tilt right | The dog focused and then head is titlted to the right side. | Duration |
| EAR ACTION DESCRIPTORS (EADs) | EAD101 | Ears Forward | The ears are turned or pushed forward (rostrally). In In dogs with pricked ears the pinna becomes rigid and tense. In dogs with floppy ears the base of the pinna is raised and pulled forward. Wrinkles may be formed along the midline frontal region. | Duration |
|  | EAD102 | Ears Adductor | The ears are adducted, the base of the pinnas become closer together, by being pulled towards the head midline. The distance between the ears decreases. A vertical wrinkle may appear on the frontal region. | Duration |
|  | EAD103 | Ears Flattener | Ears are pulled caudally, being flattened against the head.The pinna is pulled caudally, in the direction of the back of the head. In a frontal profile, the ears may disappear from the view. | Duration |
|  | EAD104  (R-L) | Ears Rotator (lateralized movement) | The ears are rotated laterally and externally. Dog can rotate both ears (EAD104) or just one of them (EAD104 L or R). The internal part of the pinna is twisted out and outward-looking. | Duration |
|  |  |  |  |  |
|  |  |  |  |  |
|  | EAD105 | Ears Downward | The ears are pulled ventrally, laterally. The base of the pinna in both ears moves away from each other. The distance between the ears increases. The frontal region skin is stretched, and the head takes a rounder shape. | Duration |

**Table 4** – General Behaviours Ethogram

| **General Category** | **Behavior** | **Description** | **Type of measure** |
| --- | --- | --- | --- |
| Position | Stand | The dog stand with all four paws on the ground and does not move. | Duration |
|  | Sit | Stationary position, with only the backside in contact with the ground. | Duration |
|  | Lay | Dog's belly touches the ground as it lays completely down on the floor. | Duration |
| Posture | High | The dog is with all four paws on the ground, the head is kept held up high while the ears are erected and held forward and the tail is kept high above the back line. Piloerection can be observed. | Duration |
|  | Neutral | The dog is with all four paws on the ground, the head is high above the back line or on the back line. The tail is kept on the same line of the back or slightly above. | Duration |
|  | Low | The dog stand (may have one of its front paws lifted), while the head is low, with ears back and flattened, disappearing behind it. The tail is down or held between the legs. | Duration |
| Locomotion | Back | The dog walks backwards. | Duration |
|  | Freeze | Reactive immobility. The body is completely rigid, while the dog keeps staring the stimuli (or the apparatus). | Duration |
|  | Still | Absence of movement, differently from freezing the dog's body is not rigid, but kept in a fixed posture. | Duration |
|  | Trot | The dog is trotting round the room. | Duration |
|  | Walking | The dog is walking. | Duration |
| Body Orientation | Door | The dog has its body directed towards the exit door. | Duration |
|  | Owner | The dog has its body directed towards the owner. | Duration |
|  | Set-up | The dog has its body directed towards the apparatus. | Duration |
|  | Stimuli | The dog has its body directed towards the stimuli. | Duration |
| Head | Face Down | The dog keeps its head downwards towards the floor. | Duration |
|  | Door | The head of the dog is directed towards the exit door. | Duration |
|  | Owner | The head of the dog is directed towards the owner. | Duration |
|  | Set-up | The head of the dog is directed towards the apparatus (excluded the stimuli window) | Duration |
|  | Stimulus | The head of the dog is directed towards the stimuli window. | Duration |
|  | Turn left | The dog is looking at the stimuli's window (food or experimenter) and then turns the head to its left. Looking away from the stimuli (food/experimenter) | Duration |
|  | Turn right | The dog is looking at the stimuli/the apparatus and then turns the head to its right. Looking away from the stimuli (food/experimenter). | Duration |
| Tail | Tail High | The tail is kept above the line of the back. | Duration |
|  | Tail Neutral | The tail is kept horizontally on the same line of the back. | Duration |
|  | Tail Down | The tail is kept below the line of the back, straight-down position, relaxed. | Duration |
|  | Tail between legs | The tail is tucked between the hind legs. | Duration |
| Wagging | Slow | The dog moves its tail back and forth in slow movements. | Duration |
|  | Fast | The dog moves its tail back and forth in fast movements. | Duration |
| Contact/Proximity | Contact with owner | The dog searches or keeps physical contact with its owner. | Duration |
|  | Door Proximity | The dog moves and remains close to the exit door. | Duration |
|  | Behind the owner | The dog sits or remains behind its owner’s chair | Duration |
| Events | Arching the Back | The dog arches its back upwards. | Duration |
|  | Autogrooming | The dog starts licking and wiping its own body in order to clean it. | Duration |
|  | Bark | Loud and short typical vocalization, which may be repetitive. | Duration |
|  | Drinking | Whenever the dog starts drinking water from the bowl. | Duration |
|  | Looking Away | The dog is looking at the stimuli window or at the experimenter (in FS) and averts its gaze ( | Event |
|  | Paw lifting | The dog lifts one of the front paws, even slightly. | Duration |
|  | Paw Tapping | The dog taps repetitively on the floor both of the front paws. | Duration |
|  | Pushing Apparatus | The dog gets closer to the apparatus and starts pushing it with its forelegs or its muzzle. | Duration |
|  | Scratching | The dog scratches repeatedly its neck or face with the hind legs. | Duration |
|  | Scratching the Door | The dog moves closer to the exit door and scratches it with is forelegs. | Duration |
|  | Shaking | Dog's whole body and head starts moving rapidly, from side to side. | Event |
|  | Sneezing | Sudden expulsion of air from the nostrils, usually accompanied by the closure of both eyes and opening of the mouth. | Duration |
|  | Sniffing Apparatus | Dog's muzzle gets closer to the apparatus, while its nose snuffles it and nostrils moves quickly. | Duration |
|  | Sniffing Environment | Dog's muzzle gets closer to the floor, the exit room, or other items inside the experimental room, while its nose snuffles it and nostrils moves quickly. | Duration |
|  | Stretching | The whole body is stretched in various ways: forelegs may be leant down while the dog's back is arched; hind legs may be straightened while the head is held up high. | Event |
|  | Tail Chasing | The dog starts circling around chasing its own tail. | Duration |
|  | Trembling | Dog’s whole body starts shivering. | Duration |
|  | Whine | The dog starts vocalizing, making persistently whines. | Duration |

**Figure 1 –** Different Ears Action Descriptors in dogs with floppy and erected ears.
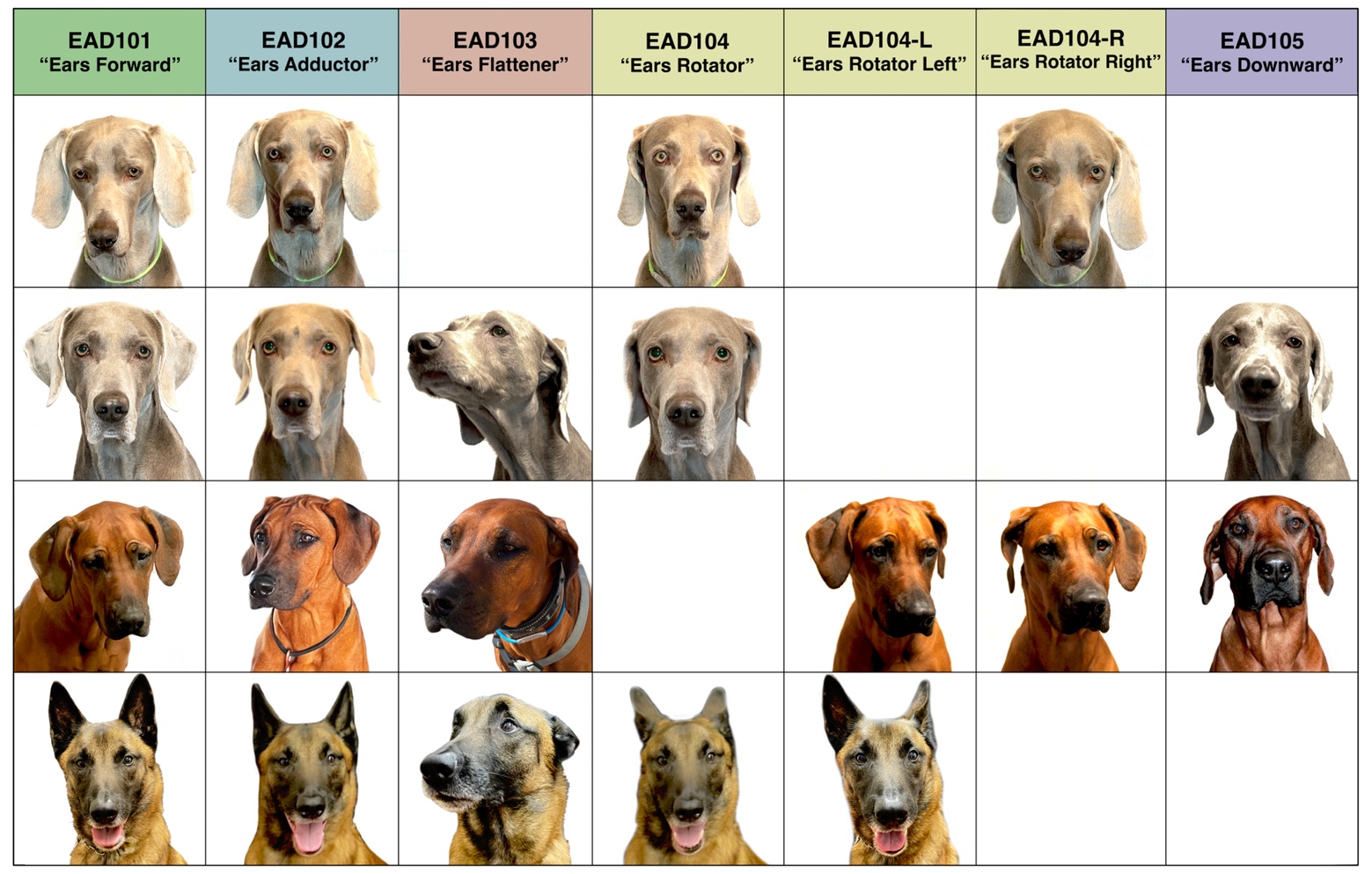


**Statistical analysis results**

Tables from 5 to 19, page 10 to 15.
Results of the generalized linear mixed effect model with the occurrence of the behavioural variables (yes/no) as a response variable and condition (frustration non-social/positive anticipation/frustration social) as test predictors, dog type (hunting type/shepherd type), subject age, subject sex (female/male), and trial as control predictors.

**Table 5 –** **AU145, Blink**. Example of full.model, null.model and trial.model used for all the behaviours to assess the effect of condition (comparison between full.model and null.model) and the effect of the trial (comparison between trial.model and full.model).

full.AU145=glmmTMB(binomial~condition+dog_type+age+sex+(1|subject), data=AU145_data,

family=binomial)

null.AU145=glmmTMB(binomial~dog_type+age+sex+(1|subject),data=AU145_data, family=binomial)

trial.AU145=glmmTMB(binomial~condition+trial+dog_type+age+sex+(1|subject), data=AU145_data, family=binomial)

|  | **Nested models** | **df** | **AIC** | **-2LL** | **Likelihood ratio test** |
| --- | --- | --- | --- | --- | --- |
| **AU145 Blink** | **Null** | 5 | 746.649 | 736.649 | -- |
|  | **Plus condition** | 7 | 728.468 | 714.181 | χ^2^_2_= 22.181, p = .000 |
|  | **Plus trial** | 11 | 720.542 | 698.542 | χ^2^_4_= 15.926, p = .003 |
| as.data.frame(anova(full.AU145, null.AU145, test= “Chisq”)  as.data.frame(anova(full.AU145, trial.AU145, test= “Chisq”) | | | | | |

| **BLINK - BINOMIAL** | **Estimate** | **Std. Error** | **OR** | **z-value** | **P-value** | **Lower CI** | **Upper  CI** | **min** | **max** |
| --- | --- | --- | --- | --- | --- | --- | --- | --- | --- |
| **(Intercept)** | -1.008 | 0.358 |  |  |  | -1.699 | -0.259 | -1.128 | -0.872 |
| **conditionFS** | 0.492 | 0.196 | 1.636 | 2.510 | 0.012 | 0.108 | 0.892 | 0.423 | 0.562 |
| **conditionPA** | -0.380 | 0.200 | 0.684 | -1.893 | 0.058 | -0.768 | -0.020 | -0.494 | -0.326 |
| **dog_typesheperdtype** | -0.060 | 0.247 | 0.941 | -0.241 | 0.809 | -0.505 | 0.451 | -0.171 | 0.006 |
| **age** | 0.047 | 0.046 | 1.048 | 1.020 | 0.308 | -0.043 | 0.137 | 0.033 | 0.067 |
| **sexmale** | -0.725 | 0.242 | 0.484 | -2.994 | 0.003 | -1.209 | -0.262 | -0.814 | -0.635 |
| **trial2** | 0.693 | 0.266 | 1.999 | 2.602 | 0.009 | 0.135 | 1.241 | 0.606 | 0.789 |
| **trial3** | 0.947 | 0.266 | 2.578 | 3.565 | 0.000 | 0.433 | 1.471 | 0.863 | 1.048 |
| **trial4** | 1.009 | 0.266 | 2.742 | 3.800 | 0.000 | 0.503 | 1.512 | 0.928 | 1.112 |
| **trial5** | 1.072 | 0.266 | 2.275 | 4.033 | 0.000 | 0.559 | 1.591 | 0.992 | 1.176 |

| **AU145, Blink – conditions (PA, FN, FS) x trial, mean ± sd** | | | | |
| --- | --- | --- | --- | --- |
| **1_PA** | **2_PA** | **3_PA** | **4_PA** | **5_PA** |
| 0.408 ± 0.643 | 0.408 ± 0.705 | 0.551 ± 0.709 | 0.306 ± 0.548 | 0.449 ± 0.679 |
| **1_FN** | **2_FN** | **3_FN** | **4_FN** | **5_FN** |
| 0.224 ± 0.550 | 0.510 ± 0.739 | 0.694 ± 0.847 | 0.612 ± 0.759 | 0.531 ± 0.544 |
| **1_FS** | **2_FS** | **3_FS** | **4_FS** | **5_FS** |
| 0.469 ± 0.892 | 0.673 ± 0.689 | 0.510 ± 0.649 | 0.939 ± 0.827 | 0.837 ± 0.825 |

**Table 6 – AU101, Inner Brow Raiser**

|  | **Nested models** | **df** | **AIC** | **-2LL** | **Likelihood ratio test** |
| --- | --- | --- | --- | --- | --- |
| **Inner Brow Raiser** | **Null** | 5 | 717.596 | 707.596 | -- |
|  | **Plus condition** | 7 | 718.295 | 704.295 | χ^2^_2_= 3.301, p = .192 |
|  | **Plus trial** | 11 | 723.682 | 701.682 | χ^2^_4_= 2.614, p = .624 |
| as.data.frame(anova(full.AU101, null.AU101, test= “Chisq”)  as.data.frame(anova(full.AU101, trial.AU101, test= “Chisq”) | | | | | |

| **AU101 - BINOMIAL** | **Estimate** | **Std. Error** | **OR** | **z-value** | **P-value** | **Lower Ci** | **Upper CI** | **min** | **max** |
| --- | --- | --- | --- | --- | --- | --- | --- | --- | --- |
| **(Intercept)** | 0.214 | 0.483 |  |  |  | -0.751 | 1.219 | 0.035 | 0.447 |
| **conditionFS** | 0.132 | 0.205 | 1.411 | 0.642 | 0.521 | -0.244 | 0.514 | 0.027 | 0.221 |
| **conditionPA** | -0.148 | 0.204 | 0.862 | -0.726 | 0.468 | -0.521 | 0.248 | -0.260 | -0.066 |
| **dog_typesheperdtype** | 0.454 | 0.371 | 1.575 | 1.222 | 0.222 | -0.299 | 1.227 | 0.293 | 0.626 |
| **age** | -0.079 | 0.069 | 0.924 | -1.134 | 0.257 | -0.225 | 0.045 | -0.113 | -0.047 |
| **sexmale** | -0.000 | 0.363 | 1 | -0.001 | 0.999 | -0.729 | 0.728 | -0.126 | 0.106 |

| **AU101, Inner Brow Raiser – conditions (PA, FN, FS) x trial, mean ± sd** | | | | |
| --- | --- | --- | --- | --- |
| **1_PA** | **2_PA** | **3_PA** | **4_PA** | **5_PA** |
| 0.588 ± 0.915 | 0.673 ± 0.793 | 0.641 ± 0.808 | 0.763 ± 1.152 | 0.824 ± 1.187 |
| **1_FN** | **2_FN** | **3_FN** | **4_FN** | **5_FN** |
| 0.816 ± 1.030 | 0.865 ± 1.079 | 0.692 ± 0.952 | 0.788 ± 1.058 | 1.175 ± 1.517 |
| **1_FS** | **2_FS** | **3_FS** | **4_FS** | **5_FS** |
| 0.751 ± 1.187 | 1.359 ± 1.468 | 1.192 ± 1.513 | 0.914 ± 1.205 | 0.992 ± 1.235 |

**Table 7 – Showing the Sclera**

|  | **Nested models** | **df** | **AIC** | **-2LL** | **Likelihood ratio test** |
| --- | --- | --- | --- | --- | --- |
| **Sclera** | **Null** | 5 | 696.492 | 686.492 | -- |
|  | **Plus condition** | 7 | 700.324 | 686.324 | χ^2^_2_= 0.168, p = .920 |
|  | **Plus trial** | 11 | 703.131 | 681.131 | χ^2^_4_= 5.193, p = .268 |
| as.data.frame(anova(full.SCLERA, null.SCLERA, test= “Chisq”)  as.data.frame(anova(full.SCLERA, trial.SCLERA, test= “Chisq”) | | | | | |

| **SCLERA - BINOMIAL** | **Estimate** | **Std. Error** | **OR** | **z-value** | **P-value** | **Lower CI** | **Upper CI** | **min** | **max** |
| --- | --- | --- | --- | --- | --- | --- | --- | --- | --- |
| **(Intercept)** | -1.162 | 0.491 |  |  |  | -2.171 | -0.284 | -2.171 | -0.284 |
| **conditionFS** | 0.064 | 0.206 | 1.066 | 0.309 | 0.757 | -0.356 | 0.451 | -0.356 | 0.451 |
| **conditionPA** | 0.106 | 0.206 | 1.111 | 0.515 | 0.607 | -0.275 | 0.495 | -0.275 | 0.495 |
| **dog_typesheperdtype** | 0.301 | 0.380 | 1.351 | 0.791 | 0.429 | -0.433 | 1.038 | -0.433 | 1.038 |
| **age** | 0.014 | 0.071 | 1.014 | 0.195 | 0.846 | -0.120 | 0.164 | -0.120 | 0.164 |
| **sexmale** | -0.004 | 0.371 | 0.996 | -0.011 | 0.991 | -0.676 | 0.742 | -0.676 | 0.742 |

| **SCLERA, Showing the Sclera – conditions (PA, FN, FS) x trial, mean ± sd** | | | | |
| --- | --- | --- | --- | --- |
| **1_PA** | **2_PA** | **3_PA** | **4_PA** | **5_PA** |
| 0.531 ± 1.029 | 0.616 ± 1.010 | 0.469 ± 0.835 | 0.535 ± 0.786 | 0.477 ± 0.795 |
| **1_FN** | **2_FN** | **3_FN** | **4_FN** | **5_FN** |
| 0.343 ± 0.713 | 0.559 ± 1.065 | 0.343 ± 0.648 | 0.441 ± 0.709 | 0.726 ± 1.270 |
| **1_FS** | **2_FS** | **3_FS** | **4_FS** | **5_FS** |
| 0.290 ± 0.736 | 0.784 ± 1.220 | 0.641 ± 1.109 | 0.612 ± 1.032 | 0.559 ± 0.847 |

**Table 8 – Nose Lick**

|  | **Nested models** | **df** | **AIC** | **-2LL** | **Likelihood ratio test** |
| --- | --- | --- | --- | --- | --- |
| **Nose lick** | **Null** | 5 | 412.153 | 402.153 | -- |
|  | **Plus condition** | 7 | 407.745 | 393.745 | χ^2^_2_= 8.407, p = .015 |
|  | **Plus trial** | 11 | 703.131 | 681.131 | χ^2^_4_= 0.905, p = .924 |
| as.data.frame(anova(full.AD137, null.AD137, test= “Chisq”)  as.data.frame(anova(full.AD137, trial.AD137, test= “Chisq”) | | | | | |

| **AD137 - BINOMIAL** | **Estimate** | **Std. Error** | **OR** | **z-value** | **P-value** | **Lower CI** | **Upper CI** | **min** | **max** |
| --- | --- | --- | --- | --- | --- | --- | --- | --- | --- |
| **(Intercept)** | -2.583 | 0.458 |  |  |  | -3.542 | -1.746 | -2.833 | -2.447 |
| **conditionFS** | 0.603 | 0.289 | 1.828 | 2.082 | 0.037 | 0.082 | 1.247 | 0.502 | 0.713 |
| **conditionPA** | 0.097 | 0.313 | 1.102 | 0.310 | 0.757 | -0.583 | 0.729 | -0.163 | 0.204 |
| **dog_typesheperdtype** | 0.109 | 0.278 | 1.115 | 0.392 | 0.695 | -0.433 | 0.668 | 0.008 | 0.224 |
| **age** | 0.049 | 0.051 | 1.050 | 0.962 | 0.336 | -0.052 | 0.154 | 0.020 | 0.066 |
| **sexmale** | -0.216 | 0.273 | 0.805 | -0.793 | 0.428 | -0.763 | 0.331 | -0.314 | -0.125 |

| **AD137, Nose Lick – conditions (PA, FN, FS) x trial, mean ± sd** | | | | |
| --- | --- | --- | --- | --- |
| **1_PA** | **2_PA** | **3_PA** | **4_PA** | **5_PA** |
| 0.102 ± 0.306 | 0.122 ± 0.484 | 0.122 ± 0.331 | 0.061 ± 0.242 | 0.143 ± 0.408 |
| **1_FN** | **2_FN** | **3_FN** | **4_FN** | **5_FN** |
| 0.061 ± 0.242 | 0.082 ± 0.277 | 0.122 ± 0.389 | 0.143 ± 0.353 | 0.082 ± 0.344 |
| **1_FS** | **2_FS** | **3_FS** | **4_FS** | **5_FS** |
| 0.184 ± 0.391 | 0.224 ± 0.468 | 0.082 ± 0.277 | 0.102 ± 0.306 | 0.184 ± 0.391 |

**Table 9 – Looking away**

|  | **Nested models** | **df** | **AIC** | **-2LL** | **Likelihood ratio test** |
| --- | --- | --- | --- | --- | --- |
| **Looking away** | **Null** | 5 | 756.615 | 746.615 | -- |
|  | **Plus condition** | 7 | 747.982 | 733.982 | χ^2^_2_= 12.632, p = .002 |
|  | **Plus trial** | 11 | 747.381 | 725.381 | χ^2^_4_= 8.602, p = .072 |
| as.data.frame(anova(full.LAWAY, null.LAWAY, test= “Chisq”)  as.data.frame(anova(full.LAWAY, trial.LAWAY, test= “Chisq”) | | | | | |

| **LOOKING AWAY - BINOMIAL** | **Estimate** | **Std. Error** | **OR** | **z-value** | **P-value** | **Lower CI** | **Upper CI** | **min** | **max** |
| --- | --- | --- | --- | --- | --- | --- | --- | --- | --- |
| **(Intercept)** | -0.874 | 0.348 |  |  |  | -1.546 | -0.212 | -1.011 | -0.713 |
| **conditionFS** | -0.147 | 0.191 | 0.863 | -0.769 | 0.442 | -0.524 | 0.216 | -0.224 | -0.076 |
| **conditionPA** | -0.764 | 0.198 | 0.466 | -3.850 | 0.000 | -1.174 | -0.372 | -0.843 | -0.701 |
| **dog_typesheperdtype** | -0.133 | 0.239 | 0.875 | -0.557 | 0.578 | -0.562 | 0.341 | -0.238 | -0.076 |
| **age** | 0.064 | 0.045 | 1.066 | 1.431 | 0.153 | -0.021 | 0.153 | 0.051 | 0.088 |
| **sexmale** | -0.076 | 0.234 | 0.927 | -0.324 | 0.746 | -0.542 | 0.374 | -0.144 | 0.014 |

| **LOOKING_AWAY – conditions (PA, FN, FS) x trial, mean ± sd** | | | | |
| --- | --- | --- | --- | --- |
| **1_PA** | **2_PA** | **3_PA** | **4_PA** | **5_PA** |
| 0.265 ± 0.490 | 0.245 ± 0.434 | 0.347 ± 0.481 | 0.408 ± 0.643 | 0.388 ± 0.492 |
| **1_FN** | **2_FN** | **3_FN** | **4_FN** | **5_FN** |
| 0.388 ± 0.492 | 0.571 ± 0.645 | 0.531 ± 0.544 | 0.490 ± 0.505 | 0.510 ± 0.544 |
| **1_FS** | **2_FS** | **3_FS** | **4_FS** | **5_FS** |
| 0.286 ± 0.500 | 0.592 ± 0.643 | 0.408 ± 0.537 | 0.571 ± 0.612 | 0.592 ± 0.609 |

**Table 10 – Wagging the tail**

|  | **Nested models** | **df** | **AIC** | **-2LL** | **Likelihood ratio test** |
| --- | --- | --- | --- | --- | --- |
| **Tail wagging** | **Null** | 5 | 443.146 | 433.146 | -- |
|  | **Plus condition** | 7 | 405.699 | 391.699 | χ^2^_2_= 41.447, p = .000 |
|  | **Plus trial** | 11 | 409.056 | 387.056 | χ^2^_4_= 4.643, p = .326 |
| as.data.frame(anova(full.WAG, null.WAG, test= “Chisq”)  as.data.frame(anova(full.WAG, trial.WAG, test= “Chisq”) | | | | | |

| **WAGGING - BINOMIAL** | **Estimate** | **Std. Error** | **OR** | **z-value** | **P-value** | **Lower CI** | **Upper CI** | **min** | **max** |
| --- | --- | --- | --- | --- | --- | --- | --- | --- | --- |
| **(Intercept)** | -3.577 | 1.344 |  |  |  | -6.601 | -1.209 | -4.121 | -3.051 |
| **conditionFS** | 2.051 | 0.334 | 7.776 | 6.134 | 0.000 | 1.371 | 2.902 | 1.898 | 2.248 |
| **conditionPA** | 0.485 | 0.318 | 1.624 | 1.525 | 0.127 | -0.182 | 1.261 | 0.249 | 0.727 |
| **dog_typesheperdtype** | -3.280 | 1.113 | 0.038 | -2.947 | 0.003 | -5.940 | -1.281 | -3.793 | -2.892 |
| **age** | 0.405 | 0.197 | 1.499 | 2.055 | 0.040 | 0.032 | 0.871 | 0.321 | 0.516 |
| **sexmale** | -1.123 | 1.015 | 0.325 | -1.107 | 0.268 | -3.384 | 0.763 | -1.511 | -0.826 |

| **WAGGING – conditions (PA, FN, FS) x trial, mean ± sd** | | | | |
| --- | --- | --- | --- | --- |
| **1_PA** | **2_PA** | **3_PA** | **4_PA** | **5_PA** |
| 1.077 ± 1.913 | 0.894 ± 1.796 | 0.763 ± 1.755 | 0.796 ± 1.754 | 0.579 ± 1.564 |
| **1_FN** | **2_FN** | **3_FN** | **4_FN** | **5_FN** |
| 0.453 ± 1.286 | 0.490 ± 1.360 | 0.535 ± 1.457 | 0.690 ± 1.608 | 0.690 ± 1.569 |
| **1_FS** | **2_FS** | **3_FS** | **4_FS** | **5_FS** |
| 1.743 ± 2.328 | 1.375 ± 2.184 | 1.216 ± 2.187 | 1.514 ± 2.176 | 1.535 ± 2.236 |

**Table 11 – Whining**

|  | **Nested models** | **df** | **AIC** | **-2LL** | **Likelihood ratio test** |
| --- | --- | --- | --- | --- | --- |
| **Whining** | **Null** | 5 | 309.117 | 299.117 | -- |
|  | **Plus condition** | 7 | 300.565 | 286.565 | χ^2^_2_= 12.552, p = .002 |
|  | **Plus trial** | 11 | 305.527 | 283.527 | χ^2^_4_= 3.037, p = .552 |
| as.data.frame(anova(full.WHINE, null.WHINE, test= “Chisq”)  as.data.frame(anova(full.WHINE, trial.WHINE, test= “Chisq”) | | | | | |

| **WHINING - BINOMIAL** | **Estimate** | **Std. Error** | **OR** | **z-value** | **Pr(>\|z\|)** | **Lower CI** | **Upper CI** | **min** | **max** |
| --- | --- | --- | --- | --- | --- | --- | --- | --- | --- |
| **(Intercept)** | -5.280 | 1.068 |  |  |  | -8.213 | -3.521 | -5.857 | -5.014 |
| **conditionFS** | 1.020 | 0.361 | 2.773 | 2.823 | 0.005 | 0.380 | 1.786 | 0.891 | 1.163 |
| **conditionPA** | -0.679 | 0.451 | 0.507 | -1.507 | 0.132 | -1.681 | 0.215 | -0.998 | -0.425 |
| **dog_typesheperdtype** | -0.383 | 0.692 | 0.681 | -0.554 | 0.579 | -1.818 | 1.086 | -0.630 | -0.073 |
| **age** | 0.085 | 0.129 | 1.089 | 0.655 | 0.513 | -0.172 | 0.349 | 0.001 | 0.140 |
| **sexmale** | 0.794 | 0.688 | 2.212 | 1.154 | 0.248 | -0.661 | 2.247 | 0.550 | 1.027 |

| **WHINING – conditions (PA, FN, FS) x trial, mean ± sd** | | | | |
| --- | --- | --- | --- | --- |
| **1_PA** | **2_PA** | **3_PA** | **4_PA** | **5_PA** |
| 0.028 ± 0.141 | 0.069 ± 0.293 | 0.061 ± 0.428 | 0.045 ± 0.159 | 0.000 ± 0.000 |
| **1_FN** | **2_FN** | **3_FN** | **4_FN** | **5_FN** |
| 0.028 ± 0.153 | 0.037 ± 0.257 | 0.016 ± 0.080 | 0.086 ± 0.305 | 0.130 ± 0.386 |
| **1_FS** | **2_FS** | **3_FS** | **4_FS** | **5_FS** |
| 0.098 ± 0.464 | 0.184 ± 0.458 | 0.147 ± 0.348 | 0.073 ± 0.307 | 0.110 ± 0.370 |

**Table 12 – Pushing the apparatus**

|  | **Nested models** | **df** | **AIC** | **-2LL** | **Likelihood ratio test** |
| --- | --- | --- | --- | --- | --- |
| **PHSH** | **Null** | 5 | 438.467 | 428.467 | -- |
|  | **Plus condition** | 7 | 418.838 | 404.838 | χ^2^_2_= 23.629, p = .000 |
|  | **Plus trial** | 11 | 415.419 | 11.418 | χ^2^_4_= 11.418, p = .022 |
| as.data.frame(anova(full.WHINE, null.WHINE, test= “Chisq”)  as.data.frame(anova(full.WHINE, trial.WHINE, test= “Chisq”) | | | | | |

| **PUSHING APPARATUS - BINOMIAL** | **Estimate** | **Std. Error** | **OR** | **z-value** | **P-value** | **Lower CI** | **Upper CI** | **min** | **max** |
| --- | --- | --- | --- | --- | --- | --- | --- | --- | --- |
| **(Intercept)** | -3.748 | 0.817 |  |  |  | -5.579 | -2.308 | -4.020 | -3.553 |
| **conditionFS** | 0.180 | 0.329 | 1.197 | 0.546 | 0.585 | -0.506 | 0.808 | -0.040 | 0.306 |
| **conditionPA** | 1.566 | 0.312 | 4.787 | 5.017 | 0.000 | 0.969 | 2.267 | 1.460 | 1.719 |
| **dog_typesheperdtype** | 0.367 | 0.585 | 1.443 | 0.627 | 0.530 | -0.814 | 1.497 | 0.022 | 0.583 |
| **age** | 0.331 | 0.113 | 1.392 | 2.929 | 0.003 | 0.111 | 0.563 | 0.297 | 0.385 |
| **sexmale** | -0.735 | 0.569 | 0.479 | -1.290 | 0.197 | -1.918 | 0.383 | -1.010 | -0.558 |
| **trial2** | -1.000 | 0.363 | 0.368 | -2.756 | 0.006 | -1.798 | -0.267 | -1.176 | -0.901 |
| **trial3** | -1.227 | 0.373 | 0.293 | -3.285 | 0.001 | -2.007 | -0.527 | -1.377 | -1.074 |
| **trial4** | -1.564 | 0.392 | 0.209 | -3.987 | 0.000 | -2.446 | -0.863 | -1.723 | -1.414 |
| **trial5** | -1.564 | 0.392 | 0.209 | -3.987 | 0.000 | -2.402 | -0.931 | -1.751 | -1.462 |

| **PUSHING_APPARATUS – conditions (PA, FN, FS) x trial, mean ± sd** | | | | |
| --- | --- | --- | --- | --- |
| **1_PA** | **2_PA** | **3_PA** | **4_PA** | **5_PA** |
| 0.408 ± 0.596 | 0.196 ± 0.512 | 0.188 ± 0.415 | 0.277 ± 0.768 | 0.257 ± 0.503 |
| **1_FN** | **2_FN** | **3_FN** | **4_FN** | **5_FN** |
| 0.163 ± 0.445 | 0.204 ± 0.780 | 0.208 ± 0.713 | 0.045 ± 0.213 | 0.000 ± 0.000 |
| **1_FS** | **2_FS** | **3_FS** | **4_FS** | **5_FS** |
| 0.208 ± 0.570 | 0.147 ± 0.412 | 0.118 ± 0.518 | 0.155 ± 0.599 | 0.045 ± 0.154 |

**Table 13 – Sniffing the environment**

|  | **Nested models** | **df** | **AIC** | **-2LL** | **Likelihood ratio test** |
| --- | --- | --- | --- | --- | --- |
| **SNIFFING** | **Null** | 5 | 251.780 | 241.780 | -- |
|  | **Plus condition** | 7 | 252.129 | 238.129 | χ^2^_2_= 3.651, p = .161 |
|  | **Plus trial** | 11 | 251.402 | 229.402 | χ^2^_4_= 8.727, p = .068 |
| as.data.frame(anova(full.SNIFFING, null.SNIFFING, test= “Chisq”)  as.data.frame(anova(full.SNIFFING, trial.SNIFFING, test= “Chisq”) | | | | | |

| **SNIFFING ENVIRONMENT - BINOMIAL** | **Estimate** | **Std. Error** | **OR** | **z value** | **P-value** | **Lower CI** | **Upper CI** | **min** | **max** |
| --- | --- | --- | --- | --- | --- | --- | --- | --- | --- |
| **(Intercept)** | -3.988 | 0.809 |  |  |  | -6.382 | -2.709 | -4.401 | -3.867 |
| **conditionFS** | -0.658 | 0.444 | 0.518 | -1.482 | 0.138 | -1.686 | 0.220 | -0.800 | -0.324 |
| **conditionPA** | -0.787 | 0.459 | 0.455 | -1.713 | 0.087 | -1.843 | 0.166 | -1.026 | -0.453 |
| **dog_typesheperdtype** | 0.026 | 0.445 | 0.974 | 0.059 | 0.953 | -0.817 | 0.992 | -0.120 | 0.214 |
| **age** | 0.066 | 0.084 | 1.068 | 0.779 | 0.436 | -0.097 | 0.236 | 0.036 | 0.107 |
| **sexmale** | 1.174 | 0.489 | 3.235 | 2.403 | 0.016 | 0.293 | 2.413 | 1.047 | 1.287 |

| **SNIFFING_ENVIRONMENT – conditions (PA, FN, FS) x trial, mean ± sd** | | | | |
| --- | --- | --- | --- | --- |
| **1_PA** | **2_PA** | **3_PA** | **4_PA** | **5_PA** |
| 0.020 ± 0.093 | 0.114 ± 0.743 | 0.000 ± 0.000 | 0.163 ± 0.836 | 0.037 ± 0.207 |
| **1_FN** | **2_FN** | **3_FN** | **4_FN** | **5_FN** |
| 0.106 ± 0.743 | 0.053 ± 0.371 | 0.184 ± 0.711 | 0.371 ± 1.028 | 0.220 ± 0.712 |
| **1_FS** | **2_FS** | **3_FS** | **4_FS** | **5_FS** |
| 0.049 ± 0.343 | 0.045 ± 0.314 | 0.122 ± 0.511 | 0.016 ± 0. 080 | 0.073 ± 0.270 |

**Table 14 – Ears position EAD101 – Ears Forward**

|  | **Nested models** | **df** | **AIC** | **-2LL** | **Likelihood ratio test** |
| --- | --- | --- | --- | --- | --- |
| **EAD101** | **Null** | 5 | 521.181 | 511.181 | -- |
|  | **Plus condition** | 7 | 510.948 | 496.948 | χ^2^_2_= 14.233, p = .001 |
|  | **Plus trial** | 11 | 501.513 | 479.513 | χ^2^_4_= 17.435, p = .002 |
| as.data.frame(anova(full.EAD101, null.EAD101, test= “Chisq”)  as.data.frame(anova(full.EAD101, trial.EAD101, test= “Chisq”) | | | | | |

| **EAD101 - BINOMIAL** | **Estimate** | **Std. Error** | **OR** | **z-value** | **P-value** | **Lower CI** | **Upper CI** | **min** | **max** |
| --- | --- | --- | --- | --- | --- | --- | --- | --- | --- |
| **(Intercept)** | 2.625 | 0.642 |  |  |  | 1.364 | 4.062 | 2.272 | 2.881 |
| **conditionFS** | 0.557 | 0.259 | 1.745 | 2.153 | 0.031 | 0.074 | 1.080 | 0.442 | 0.679 |
| **conditionPA** | 0.825 | 0.269 | 2.281 | 3.074 | 0.002 | 0.290 | 1.339 | 0.707 | 1.009 |
| **dog_typesheperdtype** | 0.558 | 0.478 | 1.747 | 1.168 | 0.243 | -0.360 | 1.587 | 0.347 | 0.709 |
| **age** | 0.013 | 0.092 | 1.013 | 0.147 | 0.883 | -0.170 | 0.201 | -0.019 | 0.070 |
| **sexmale** | -0.921 | 0.477 | 0.398 | -1.932 | 0.053 | -1.972 | 0.042 | -1.137 | -0.786 |
| **trial2** | -0.554 | 0.402 | 0.575 | -1.378 | 0.168 | -1.403 | 0.273 | -0.689 | -0.420 |
| **trial3** | -1.161 | 0.386 | 0.313 | -3.011 | 0.003 | -2.035 | -0.458 | -1.381 | -1.058 |
| **trial4** | -1.469 | 0.381 | 0.230 | -3.854 | 0.000 | -2.339 | -0.762 | -1.645 | -1.379 |
| **trial5** | -1.613 | 0.380 | 0.199 | -4.246 | 0.000 | -2.406 | -0.949 | -1.843 | -1.527 |

| **EAD101, Ears Forward – conditions (PA, FN, FS) x trial, mean ± sd** | | | | |
| --- | --- | --- | --- | --- |
| **1_PA** | **2_PA** | **3_PA** | **4_PA** | **5_PA** |
| 2.616 ± 1.923 | 2.931 ± 1.878 | 3.155 ± 1.949 | 2.837 ± 1.895 | 2.690 ± 1.940 |
| **1_FN** | **2_FN** | **3_FN** | **4_FN** | **5_FN** |
| 3.175 ± 1.882 | 2.633 ± 1.956 | 2.482 ± 2.061 | 2.286 ± 2.067 | 1.853 ± 2.019 |
| **1_FS** | **2_FS** | **3_FS** | **4_FS** | **5_FS** |
| 3.706 ± 1.705 | 3.106 ± 1.927 | 2.588 ± 2.035 | 2.457 ± 2.063 | 2.571 ± 2.066 |

**Table 15 – Ears Position EAD102 – Ears Adductor**

|  | **Nested models** | **df** | **AIC** | **-2LL** | **Likelihood ratio test** |
| --- | --- | --- | --- | --- | --- |
| **EAD102** | **Null** | 5 | 748.660 | 738.660 | -- |
|  | **Plus condition** | 7 | 747.730 | 733.730 | χ^2^_2_= 5.947, p = .203 |
|  | **Plus trial** | 11 | 749.782 | 727.782 | χ^2^_4_= 4.931, p = .085 |
| as.data.frame(anova(full.EAD102, null.EAD102, test= “Chisq”)  as.data.frame(anova(full.EAD102, trial.EAD102, test= “Chisq”) | | | | | |

| **EAD102 - BINOMIAL** | **Estimate** | **Std. Error** | **OR** | **z-value** | **P-value** | **Lower CI** | **Upper CI** | **min** | **max** |
| --- | --- | --- | --- | --- | --- | --- | --- | --- | --- |
| **(Intercept)** | -0.536 | 0.429 |  |  |  | -1.411 | 0.341 | -0.659 | -0.385 |
| **conditionFS** | -0.440 | 0.201 | 0.644 | -2.188 | 0.029 | -0.847 | -0.062 | -0.534 | -0.368 |
| **conditionPA** | -0.040 | 0.199 | 0.960 | -0.199 | 0.842 | -0.426 | 0.361 | -0.121 | 0.060 |
| **dog_typesheperdtype** | 0.085 | 0.323 | 0.918 | 0.263 | 0.793 | -0.539 | 0.715 | -0.027 | 0.215 |
| **age** | 0.008 | 0.060 | 1.008 | 0.126 | 0.900 | -0.116 | 0.130 | -0.017 | 0.033 |
| **sexmale** | -0.208 | 0.316 | 0.812 | -0.657 | 0.511 | -0.867 | 0.422 | -0.336 | -0.120 |

| **EAD102, Ears Adductor – conditions (PA, FN, FS) x trial, mean ± sd** | | | | |
| --- | --- | --- | --- | --- |
| **1_PA** | **2_PA** | **3_PA** | **4_PA** | **5_PA** |
| 1.122 ± 1.669 | 0.882 ± 1.361 | 0.959 ± 1.488 | 1.028 ± 1.487 | 1.118 ± 1.534 |
| **1_FN** | **2_FN** | **3_FN** | **4_FN** | **5_FN** |
| 0.745 ± 1.252 | 0.877 ± 1.306 | 1.057 ± 1.488 | 1.298 ± 1.825 | 1.453 ± 1.604 |
| **1_FS** | **2_FS** | **3_FS** | **4_FS** | **5_FS** |
| 0.506 ± 1.063 | 0.702 ± 1.076 | 0.975 ± 1.288 | 0.886 ± 1.433 | 0.767 ± 1.394 |

**Table 16 – Ears position EAD104 – Ears Rotator**

|  | **Nested models** | **df** | **AIC** | **-2LL** | **Likelihood ratio test** |
| --- | --- | --- | --- | --- | --- |
| **EAD102** | **Null** | 5 | 737.305 | 727.305 | -- |
|  | **Plus condition** | 7 | 737.546 | 723.546 | χ^2^_2_= 3.759, p = .153 |
|  | **Plus trial** | 11 | 738.361 | 716.361 | χ^2^_4_= 7.185, p = .126 |
| as.data.frame(anova(full.EAD104, null.EAD104, test= “Chisq”)  as.data.frame(anova(full.EAD104, trial.EAD104, test= “Chisq”) | | | | | |

| **EAD104 - BINOMIAL** | **Estimate** | **Std. Error** | **OR** | **z-value** | **P-value** | **Lower  CI** | **Upper CI** | **min** | **max** |
| --- | --- | --- | --- | --- | --- | --- | --- | --- | --- |
| **(Intercept)** | -0.544 | 0.398 |  |  |  | -1.358 | 0.214 | -0.682 | -0.349 |
| **conditionFS** | -0.257 | 0.201 | 0.773 | -1.279 | 0.201 | -0.666 | 0.137 | -0.344 | -0.201 |
| **conditionPA** | -0.321 | 0.202 | 0.725 | -1.590 | 0.112 | -0.703 | 0.066 | -0.392 | -0.225 |
| **dog_typesheperdtype** | 0.535 | 0.290 | 1.707 | 1.844 | 0.065 | -0.037 | 1.126 | 0.436 | 0.628 |
| **age** | 0.098 | 0.055 | 1.103 | 1.782 | 0.075 | -0.003 | 0.207 | 0.076 | 0.116 |
| **sexmale** | 0.191 | 0.285 | 1.210 | 0.669 | 0.504 | -0.376 | 0.711 | 0.099 | 0.268 |

| **EAD104, Ears Rotator – conditions (PA, FN, FS) x trial, mean ± sd** | | | | |
| --- | --- | --- | --- | --- |
| **1_PA** | **2_PA** | **3_PA** | **4_PA** | **5_PA** |
| 0.947 ± 1.195 | 0.979 ± 1.398 | 0.759 ± 0.887 | 0.747 ± 1.083 | 0.951 ± 1.232 |
| **1_FN** | **2_FN** | **3_FN** | **4_FN** | **5_FN** |
| 0.882 ± 1.206 | 1.261 ± 1.292 | 1.253 ± 1.520 | 1.175 ± 1.378 | 1.298 ± 1.373 |
| **1_FS** | **2_FS** | **3_FS** | **4_FS** | **5_FS** |
| 0.620 ± 1.008 | 0.832 ± 1.137 | 0.967 ± 1.137 | 1.053 ± 1.311 | 0.143 ± 1.370 |

**Table 17 – Ears position EAD105 – Ears Downward**

|  | **Nested models** | **df** | **AIC** | **-2LL** | **Likelihood ratio test** |
| --- | --- | --- | --- | --- | --- |
| **EAD102** | **Null** | 5 | 471.857 | 461.857 | -- |
|  | **Plus condition** | 7 | 472.414 | 458.414 | χ^2^_2_= 3.443, p = .179 |
|  | **Plus trial** | 11 | 474.911 | 452.911 | χ^2^_4_= 5.504, p = .239 |
| as.data.frame(anova(full.EAD105, null.EAD105, test= “Chisq”)  as.data.frame(anova(full.EAD105, trial.EAD105, test= “Chisq”) | | | | | |

| **EAD105 - BINOMIAL** | **Estimate** | **Std. Error** | **OR** | **z-value** | **P-value** | **Lower CI** | **Upper CI** | **min** | **max** |
| --- | --- | --- | --- | --- | --- | --- | --- | --- | --- |
| **(Intercept)** | -2.782 | 0.630 |  |  |  | -4.077 | -1.603 | -3.049 | -2.598 |
| **conditionFS** | 0.559 | 0.261 | 1.749 | 2.142 | 0.032 | 0.064 | 1.123 | 0.441 | 0.765 |
| **conditionPA** | 0.370 | 0.264 | 1.447 | 1.405 | 0.160 | -0.132 | 0.925 | 0.276 | 0.456 |
| **dog_typesheperdtype** | -1.493 | 0.484 | 0.225 | -3.088 | 0.002 | -2.641 | -0.637 | -1.674 | -1.366 |
| **age** | 0.219 | 0.091 | 1.245 | 2.410 | 0.016 | 0.049 | 0.403 | 0.180 | 0.266 |
| **sexmale** | 0.159 | 0.457 | 1.172 | 0.349 | 0.727 | -0.765 | 1.072 | -0.027 | 0.312 |

| **EAD105, Ears Downward – conditions (PA, FN, FS) x trial, mean ± sd** | | | | |
| --- | --- | --- | --- | --- |
| **1_PA** | **2_PA** | **3_PA** | **4_PA** | **5_PA** |
| 0.408 ± 1.086 | 0.294 ± 0.704 | 0.216 ± 0.556 | 0.412 ± 0.988 | 0.343 ± 0.762 |
| **1_FN** | **2_FN** | **3_FN** | **4_FN** | **5_FN** |
| 0.371 ± 0.861 | 0.302 ± 0.727 | 0.228 ± 0.580 | 0.294 ± 0.963 | 0.522 ± 1.262 |
| **1_FS** | **2_FS** | **3_FS** | **4_FS** | **5_FS** |
| 0.220 ± 0.613 | 0.441 ± 0.846 | 0.469 ± 1.162 | 0.661 ± 1.509 | 0.490 ± 1.177 |

**Table 18 – Ears Position EAD103 – Ears Flattener**

|  | **Nested models** | **df** | **AIC** | **-2LL** | **Likelihood ratio test** |
| --- | --- | --- | --- | --- | --- |
| **EAD102** | **Null** | 5 | 217.303 | 207.303 | -- |
|  | **Plus condition** | 7 | 212.937 | 198.937 | χ^2^_2_= 8.366, p = .015 |
|  | **Plus trial** | 11 | 218.827 | 196.827 | χ^2^_4_= 2.110, p = .716 |
| as.data.frame(anova(full.EAD103, null.EAD103, test= “Chisq”)  as.data.frame(anova(full.EAD103, trial.EAD103, test= “Chisq”) | | | | | |

| **EAD103 - BINOMIAL** | **Estimate** | **Std. Error** | **OR** | **z-value** | **P-value** | **Lower CI** | **Upper CI** | **min** | **max** |
| --- | --- | --- | --- | --- | --- | --- | --- | --- | --- |
| **(Intercept)** | -9.054 | 1.562 |  |  |  | -14.938 | -6.690 | -9.950 | -7.185 |
| **conditionFS** | 1.267 | 0.478 | 3.550 | 2.651 | 0.008 | 0.460 | 2.330 | 1.103 | 1.654 |
| **conditionPA** | -0.016 | 0.543 | 0.984 | -0.030 | 0.976 | -1.092 | 1.006 | -0.226 | 0.142 |
| **dog_typesheperdtype** | 1.850 | 0.826 | 6.359 | 2.241 | 0.025 | 0.290 | 3.820 | 1.408 | 2.248 |
| **age** | 0.374 | 0.148 | 1.453 | 2.530 | 0.011 | 0.092 | 0.745 | 0.218 | 0.455 |
| **sexmale** | 2.418 | 0.850 | 11.223 | 2.844 | 0.004 | 0.891 | 4.761 | 1.794 | 2.780 |

| **EAD103, Ears Flattener – conditions (PA, FN, FS) x trial, mean ± sd** | | | | |
| --- | --- | --- | --- | --- |
| **1_PA** | **2_PA** | **3_PA** | **4_PA** | **5_PA** |
| 0.0612 ± 0.375 | 0.033 ± 0.201 | 0.061 ± 0.332 | 0.057 ± 0.346 | 0.090 ± 0.368 |
| **1_FN** | **2_FN** | **3_FN** | **4_FN** | **5_FN** |
| 0.037 ± 0.190 | 0.102 ± 0.503 | 0.143 ± 0.594 | 0.143 ± 0.729 | 0.053 ± 0.371 |
| **1_FS** | **2_FS** | **3_FS** | **4_FS** | **5_FS** |
| 0.171 ± 0.582 | 0.090 ± 0.339 | 0.171 ± 0.752 | 0.098 ± 0.428 | 0.200 ± 0.896 |

**CORTISOL RESULTS**

Tables from 19 to 32, page 15 to 21.
Results of the generalized linear mixed effect model with the sum of the duration/frequencies of the behavioural variables in the 5 trials as a response variable. Pre_test saliva cortisol concentration as test predictor. Test condition (frustration non-social/positive anticipation/frustration social), post-test cortisol concentrations, dog type (hunting type/shepherd type), age, sex (female/male) as control predictors.

Table 19 - EAD101 – Ears Forward

|  | **Nested models** | **df** | **AIC** | **-2LL** | **Likelihood ratio test** |
| --- | --- | --- | --- | --- | --- |
| **EAD101** | **Null** | 10 | 770.692 | 750.692 | -- |
|  | **Plus pre-cortisol** | 11 | 765.779 | 743.779 | χ^2^_2_= 6.913, p = .009 |
| as.data.frame(anova(full.EAD101, null.EAD101, test= “Chisq”) | | | | | |

| **EAD101 - DURATION** | **Estimate** | **Std. Error** | **OR** | **z-value** | **P-value** | **Lower CI** | **Upper CI** | **min** | **max** |
| --- | --- | --- | --- | --- | --- | --- | --- | --- | --- |
| **(Intercept)** | 18.362 | 2.365 |  | 7.765 |  |  | 23.251 | 17.013 | 20.869 |
| **pre_c** | -1.725 | 0.647 | 0.178 | -2.668 | 0.008 | -2.937 | -0.474 | -2.306 | -1.179 |
| **conditionFS** | 2.689 | 1.199 | 14.717 | 2.243 | 0.025 | 0.331 | 4.883 | 1.856 | 3.634 |
| **conditionPA** | 2.745 | 1.192 | 15.564 | 2.303 | 0.021 | 0.383 | 5.311 | 1.953 | 3.448 |
| **post_c** | 0.612 | 0.453 | 1.844 | 1.351 | 0.177 | -0.273 | 1.509 | -0.435 | 0.837 |
| **dog_typesheperdtype** | 1.959 | 1.864 | 7.092 | 1.051 | 0.293 | -2.037 | 5.876 | 0.525 | 2.951 |
| **age** | -0.707 | 0.338 | 0.493 | -2.091 | 0.037 | -1.348 | -0.087 | -0.927 | -0.526 |
| **sexmale** | -3.137 | 1.877 | 0.043 | -1.671 | 0.095 | -6.955 | 0.396 | -4.081 | -1.476 |

Table 20 - EAD102 – Ears Adductor

|  | **Nested models** | **df** | **AIC** | **-2LL** | **Likelihood ratio test** |
| --- | --- | --- | --- | --- | --- |
| **EAD102** | **Null** | 10 | 671.553 | 651.553 | -- |
|  | **Plus pre-cortisol** | 11 | 671.792 | 649.792 | χ^2^_2_= 1.761, p = .009 |
| as.data.frame(anova(full.EAD102, null.EAD102, test= “Chisq”) | | | | | |

| **EAD102 – DURATION** | **Estimate** | **Std. Error** | **OR** | **z-value** | **P-value** | **Lower CI** | **Upper CI** | **min** | **max** |
| --- | --- | --- | --- | --- | --- | --- | --- | --- | --- |
| **(Intercept)** | 3.830 | 1.354 |  |  |  | 3.227 | 5.854 | 2.620 | 4.317 |
| **pre_c** | 0.560 | 0.422 | 1.750 | 1.327 | 0.184 | -0.450 | 0.140 | 0.103 | 0.816 |
| **conditionFS** | -1.653 | 0.870 | 0.191 | -1.899 | 0.058 | -1.457 | 0.583 | -2.284 | -0.856 |
| **conditionPA** | -0.869 | 0.866 | 0.419 | -1.002 | 0.316 | -0.883 | 0.859 | -1.292 | 0.258 |
| **post_c** | -0.169 | 0.291 | 0.845 | -0.581 | 0.561 | -0.134 | 0.276 | -0.303 | 0.926 |
| **dog_typesheperdtype** | -1.823 | 1.033 | 0.161 | -1.765 | 0.078 | -0.313 | 1.240 | -2.348 | -0.676 |
| **age** | 0.314 | 0.187 | 1.369 | 1.680 | 0.093 | -0.077 | 0.090 | 0.145 | 0.392 |
| **sexmale** | 0.445 | 1.047 | 1.560 | 0.425 | 0.671 | -0.627 | 0.801 | -0.166 | 0.978 |

Table 21 - EAD104 – Ears Rotator

|  | **Nested models** | **df** | **AIC** | **-2LL** | **Likelihood ratio test** |
| --- | --- | --- | --- | --- | --- |
| **EAD102** | **Null** | 10 | 650.813 | 630.813 | -- |
|  | **Plus pre-cortisol** | 11 | 651.616 | 629.616 | χ^2^_2_= 1.197, p = .274 |
| as.data.frame(anova(full.EAD104, null.EAD104, test= “Chisq”) | | | | | |

| **EAD104 - DURATION** | **Estimate** | **Std. Error** | **OR** | **z-value** | **P-value** | **Lower CI** | **Upper CI** | **min** | **max** |
| --- | --- | --- | --- | --- | --- | --- | --- | --- | --- |
| **(Intercept)** | 4.495 | 1.229 |  |  |  | 2.104 | 6.932 | 2.466 | 5.530 |
| **pre_c** | 0.428 | 0.388 | 1.534 | 1.101 | 0.271 | -0.312 | 1.211 | 0.166 | 0.639 |
| **conditionFS** | -1.512 | 0.800 | 0.220 | -1.890 | 0.059 | -3.060 | 0.023 | -1.976 | -1.127 |
| **conditionPA** | -1.763 | 0.796 | 0.172 | -2.214 | 0.027 | -3.303 | -0.146 | -2.163 | -1.315 |
| **post_c** | -0.308 | 0.263 | 0.735 | -1.171 | 0.242 | -0.852 | 0.176 | -0.417 | -0.026 |
| **dog_typesheperdtype** | 1.048 | 0.936 | 2.852 | 1.119 | 0.263 | -0.729 | 2.927 | 0.265 | 2.567 |
| **age** | 0.119 | 0.169 | 1.126 | 0.701 | 0.483 | -0.205 | 0.469 | -0.030 | 0.320 |
| **sexmale** | 1.068 | 0.946 | 2.909 | 1.129 | 0.259 | -0.863 | 2.888 | 0.331 | 1.698 |

Table 22 - EAD105 – Ears Downward

|  | **Nested models** | **df** | **AIC** | **-2LL** | **Likelihood ratio test** |
| --- | --- | --- | --- | --- | --- |
| **EAD102** | **Null** | 10 | 553.831 | 533.831 | -- |
|  | **Plus pre-cortisol** | 11 | 545.703 | 523.703 | χ^2^_2_= 10.128, p = .001 |
| as.data.frame(anova(full.EAD105, null.EAD105, test= “Chisq”) | | | | | |

| **EAD105 - DURATION** | **Estimate** | **Std. Error** | **OR** | **z-value** | **P-value** | **Lower CI** | **Upper CI** | **min** | **max** |
| --- | --- | --- | --- | --- | --- | --- | --- | --- | --- |
| **(Intercept)** | 0.726 | 0.952 |  |  |  | -1.174 | 2.561 | -0.216 | 1.931 |
| **pre_c** | 0.879 | 0.267 | 2.408 | 3.295 | 0.001 | 0.406 | 1.400 | 0.313 | 1.532 |
| **conditionFS** | 0.227 | 0.463 | 1.255 | 0.490 | 0.624 | -0.714 | 1.218 | -0.031 | 0.512 |
| **conditionPA** | 0.035 | 0.458 | 1.036 | 0.076 | 0.939 | -0.881 | 0.966 | -0.188 | 0.242 |
| **post_c** | -0.226 | 0.206 | 0.798 | -1.102 | 0.271 | -0.569 | 0.132 | -0.417 | -0.072 |
| **dog_typesheperdtype** | -2.231 | 0.650 | 0.107 | -3.434 | 0.001 | -3.478 | -1.000 | -2.769 | -1.357 |
| **age** | 0.019 | 0.128 | 1.019 | 0.152 | 0.879 | -0.239 | 0.258 | -0.120 | 0.122 |
| **sexmale** | 1.327 | 0.740 | 3.769 | 1.792 | 0.073 | -0.148 | 2.780 | 0.448 | 1.622 |

Table 23 - EAD103 – Ears Flattener

|  | **Nested models** | **df** | **AIC** | **-2LL** | **Likelihood ratio test** |
| --- | --- | --- | --- | --- | --- |
| **EAD103** | **Null** | 10 | 436.755 | 416.755 | -- |
|  | **Plus pre-cortisol** | 11 | 437.462 | 415.462 | χ^2^_2_= 1.293, p = .255 |
| as.data.frame(anova(full.EAD103, null.EAD103, test= “Chisq”) | | | | | |

| **EAD103 - DURATION** | **Estimate** | **Std. Error** | **OR** | **z-value** | **P-value** | **Lower CI** | **Upper CI** | **min** | **max** |
| --- | --- | --- | --- | --- | --- | --- | --- | --- | --- |
| **(Intercept)** | -1.288 | 0.787 |  |  |  | -3.000 | 0.362 | -1.735 | -0.481 |
| **pre_c** | -0.168 | 0.145 | 0.845 | -1.159 | 0.247 | -0.440 | 0.120 | -0.240 | -0.004 |
| **conditionFS** | 0.301 | 0.234 | 1.351 | 1.286 | 0.198 | -0.147 | 0.769 | 0.008 | 0.447 |
| **conditionPA** | -0.259 | 0.232 | 0.772 | -1.116 | 0.264 | -0.712 | 0.219 | -0.333 | -0.060 |
| **post_c** | -0.035 | 0.108 | 0.966 | -0.323 | 0.747 | -0.248 | 0.184 | -0.053 | 0.023 |
| **dog_typesheperdtype** | 0.609 | 0.646 | 1.838 | 0.944 | 0.345 | -0.617 | 1.897 | 0.339 | 0.814 |
| **age** | 0.247 | 0.118 | 1.280 | 2.100 | 0.036 | -0.010 | 0.469 | 0.050 | 0.333 |
| **sexmale** | 1.080 | 0.641 | 2.945 | 1.684 | 0.092 | -0.185 | 2.436 | 0.410 | 1.527 |

Table 24 - AU101 – Inner Brow Raiser

|  | **Nested models** | **df** | **AIC** | **-2LL** | **Likelihood ratio test** |
| --- | --- | --- | --- | --- | --- |
| **AU101** | **Null** | 10 | 637.446 | 617.446 | -- |
|  | **Plus pre-cortisol** | 11 | 639.425 | 617.425 | χ^2^_2_= 0.021, p = .884 |
| as.data.frame(anova(full.AU101, null.AU101, test= “Chisq”) | | | | | |

| **AU101 - DURATION** | **Estimate** | **Std. Error** | **OR** | **z-value** | **Pr(>\|z\|)** | **Lower CI** | **Upper CI** | **min** | **max** |
| --- | --- | --- | --- | --- | --- | --- | --- | --- | --- |
| **(Intercept)** | 4.889 | 1.175 |  |  |  | 2.798 | 5.121 | 3.775 | 5.534 |
| **pre_c** | 0.053 | 0.363 | 1.054 | 0.146 | 0.884 | -0.246 | 0.300 | -0.171 | 0.469 |
| **conditionFS** | 1.186 | 0.755 | 3.273 | 1.572 | 0.116 | -0.754 | 0.894 | -0.001 | 1.655 |
| **conditionPA** | -0.783 | 0.751 | 0.457 | -1.042 | 0.297 | -0.565 | 0.874 | -1.105 | -0.416 |
| **post_c** | -0.122 | 0.251 | 0.885 | -0.487 | 0.626 | -0.122 | 0.255 | -0.466 | 0.246 |
| **dog_typesheperdtype** | -0.092 | 0.897 | 0.912 | -0.103 | 0.918 | -0.535 | 0.829 | -0.760 | 0.515 |
| **age** | -0.207 | 0.162 | 0.813 | -1.280 | 0.201 | -0.096 | 0.058 | -0.298 | -0.109 |
| **sexmale** | 0.769 | 0.907 | 2.157 | 0.848 | 0.397 | -0.579 | 0.707 | 0.265 | 1.548 |

Table 25 - AU145 – Blink

|  | **Nested models** | **df** | **AIC** | **-2LL** | **Likelihood ratio test** |
| --- | --- | --- | --- | --- | --- |
| **AU145** | **Null** | 10 | 447.254 | 429.254 | -- |
|  | **Plus pre-cortisol** | 11 | 448.909 | 428.909 | χ^2^_2_= 0.345, p = .557 |
| as.data.frame(anova(full.AU145, null.AU145, test= “Chisq”) | | | | | |

| **BLINK - FREQUENCIES** | **Estimate** | **Std. Error** | **OR** | **z-value** | **P-value** | **Lower CI** | **Upper CI** | **min** | **max** |
| --- | --- | --- | --- | --- | --- | --- | --- | --- | --- |
| **(Intercept)** | 1.056 | 0.242 |  |  |  | 0.644 | 1.156 | 0.760 | 1.230 |
| **pre_c** | 0.046 | 0.078 | 1.047 | 0.593 | 0.553 | -0.081 | 0.058 | -0.033 | 0.139 |
| **conditionFS** | 0.287 | 0.133 | 1.332 | 2.163 | 0.031 | -0.246 | 0.087 | 0.189 | 0.379 |
| **conditionPA** | -0.379 | 0.157 | 0.685 | -2.417 | 0.016 | -0.208 | 0.115 | -0.524 | -0.285 |
| **post_c** | -0.100 | 0.066 | 0.905 | -1.520 | 0.129 | -0.004 | 0.084 | -0.148 | 0.006 |
| **dog_typesheperdtype** | -0.151 | 0.186 | 0.859 | -0.811 | 0.418 | -0.081 | 0.212 | -0.247 | -0.052 |
| **age** | 0.039 | 0.034 | 1.039 | 1.151 | 0.250 | -0.018 | 0.014 | 0.022 | 0.077 |
| **sexmale** | -0.405 | 0.185 | 0.667 | -2.185 | 0.029 | -0.147 | 0.159 | -0.491 | -0.223 |

Table 26 - SCLERA – Showing the sclera

|  | **Nested models** | **df** | **AIC** | **-2LL** | **Likelihood ratio test** |
| --- | --- | --- | --- | --- | --- |
| **SCLERA** | **Null** | 10 | 577.101 | 557.101 | -- |
|  | **Plus pre-cortisol** | 11 | 577.090 | 555.090 | χ^2^_2_= 2.012, p = .156 |
| as.data.frame(anova(full.SCLERA, null.SCLERA, test= “Chisq”) | | | | | |

| **SCLERA-FREQUENCY** | **Estimate** | **Std. Error** | **OR** | **z-value** | **P-value** | **Lower CI** | **Upper CI** | **min** | **max** |
| --- | --- | --- | --- | --- | --- | --- | --- | --- | --- |
| **(Intercept)** | 1.321 | 0.993 |  |  |  | 1.465 | 3.419 | 0.567 | 1.968 |
| **pre_c** | 0.405 | 0.284 | 1.499 | 1.426 | 0.154 | -0.127 | 0.270 | 0.183 | 0.582 |
| **conditionFS** | -0.069 | 0.537 | 0.933 | -0.128 | 0.898 | -0.730 | 0.709 | -0.402 | 0.378 |
| **conditionPA** | 0.067 | 0.534 | 1.069 | 0.125 | 0.901 | -0.593 | 0.570 | -0.223 | 0.514 |
| **post_c** | -0.189 | 0.198 | 0.828 | -0.959 | 0.337 | -0.166 | 0.099 | -0.296 | -0.133 |
| **dog_typesheperdtype** | 0.340 | 0.778 | 1.405 | 0.438 | 0.662 | -0.672 | 0.375 | -0.278 | 0.755 |
| **age** | 0.040 | 0.141 | 1.040 | 0.287 | 0.774 | -0.054 | 0.064 | -0.023 | 0.159 |
| **sexmale** | 0.552 | 0.783 | 1.737 | 0.705 | 0.481 | -0.615 | 0.356 | -0.325 | 1.415 |

Table 27 – AD137 - NOSE LICK

|  | **Nested models** | **df** | **AIC** | **-2LL** | **Likelihood ratio test** |
| --- | --- | --- | --- | --- | --- |
| **AD137** | **Null** | 9 | 253.589 | 235.589 | -- |
|  | **Plus pre-cortisol** | 10 | 255.586 | 235.586 | χ^2^_2_= 0.003, p = .960 |
| as.data.frame(anova(full.AD137, null.AD137, test= “Chisq”) | | | | | |

| **Nose lick - FREQUENCY** | **Estimate** | **Std. Error** | **OR** | **z-value** | **P-value** | **Lower CI** | **Upper CI** | **min** | **max** |
| --- | --- | --- | --- | --- | --- | --- | --- | --- | --- |
| **(Intercept)** | -1.136 | 0.440 |  |  |  | -2.129 | -0.301 | -1.456 | -0.862 |
| **pre_c** | -0.006 | 0.128 | 0.994 | -0.050 | 0.960 | -0.291 | 0.258 | -0.188 | 0.093 |
| **conditionFS** | 0.721 | 0.305 | 2.056 | 2.359 | 0.018 | 0.153 | 1.422 | 0.482 | 0.901 |
| **conditionPA** | 0.168 | 0.340 | 1.183 | 0.496 | 0.620 | -0.467 | 0.948 | -0.134 | 0.376 |
| **post_c** | -0.024 | 0.099 | 0.976 | -0.247 | 0.805 | -0.316 | 0.123 | -0.079 | 0.112 |
| **dog_typesheperdtype** | 0.069 | 0.293 | 1.071 | 0.236 | 0.814 | -0.509 | 0.696 | -0.233 | 0.262 |
| **age** | 0.048 | 0.052 | 1.049 | 0.909 | 0.364 | -0.061 | 0.149 | 0.012 | 0.088 |
| **sexmale** | -0.077 | 0.294 | 0.925 | -0.263 | 0.793 | -0.693 | 0.523 | -0.232 | 0.170 |

Table 28 - LOOKING AWAY

|  | **Nested models** | **df** | **AIC** | **-2LL** | **Likelihood ratio test** |
| --- | --- | --- | --- | --- | --- |
| **LOOK AWAY** | **Null** | 9 | 412.954 | 394.954 | -- |
|  | **Plus pre-cortisol** | 10 | 408.984 | 388.984 | χ^2^_2_= 5.970, p = . 0.015 |
| as.data.frame(anova(full.LAWAY, null.LAWAY, test= “Chisq”) | | | | | |

| **LOOKING AWAY - FREQUENCY** | **Estimate** | **Std. Error** | **OR** | **z-value** | **P-value** | **Lower CI** | **Upper CI** | **min** | **max** |
| --- | --- | --- | --- | --- | --- | --- | --- | --- | --- |
| **(Intercept)** | 0.439 | 0.230 |  |  |  | -0.019 | 0.865 | 0.159 | 0.565 |
| **pre_c** | 0.152 | 0.060 | 1.164 | 2.510 | 0.012 | 0.037 | 0.280 | 0.122 | 0.188 |
| **conditionFS** | -0.077 | 0.151 | 0.926 | -0.506 | 0.613 | -0.376 | 0.201 | -0.156 | -0.017 |
| **conditionPA** | -0.426 | 0.165 | 0.653 | -2.591 | 0.010 | -0.773 | -0.100 | -0.498 | -0.380 |
| **post_c** | 0.011 | 0.045 | 1.011 | 0.238 | 0.812 | -0.089 | 0.087 | -0.001 | 0.037 |
| **dog_typesheperdtype** | -0.252 | 0.151 | 0.778 | -1.671 | 0.095 | -0.521 | 0.030 | -0.355 | -0.143 |
| **age** | 0.036 | 0.028 | 1.036 | 1.259 | 0.208 | -0.023 | 0.090 | 0.018 | 0.058 |
| **sexmale** | 0.161 | 0.194 | 0.851 | 0.832 | 0.405 | -0.152 | 0.508 | -0.084 | 0.272 |

Table 29 - WAGGING

|  | **Nested models** | **df** | **AIC** | **-2LL** | **Likelihood ratio test** |
| --- | --- | --- | --- | --- | --- |
| **WAGGING** | **Null** | 10 | 763.889 | 743.889 | -- |
|  | **Plus pre-cortisol** | 11 | 763.609 | 741.609 | χ^2^_2_= 2.281, p = .131 |
| as.data.frame(anova(full.WAGGING, null.WAGGING, test= “Chisq”) | | | | | |

| **WAGGING - DURATION** | **Estimate** | **Std. Error** | **OR** | **z-value** | **P-value** | **Lower CI** | **Upper CI** | **min** | **max** |
| --- | --- | --- | --- | --- | --- | --- | --- | --- | --- |
| **(Intercept)** | 2.275 | 2.569 |  |  |  | -3.085 | 7.495 | 0.855 | 4.084 |
| **pre_c** | 0.962 | 0.634 | 2.617 | 1.518 | 0.129 | -0.244 | 2.120 | -0.817 | 1.415 |
| **conditionFS** | 4.662 | 1.124 | 105.847 | 4.147 | 0.000 | 2.461 | 6.919 | 4.021 | 5.540 |
| **conditionPA** | 1.879 | 1.118 | 6.547 | 1.681 | 0.093 | -0.365 | 4.032 | 0.855 | 2.543 |
| **post_c** | -0.469 | 0.461 | 0.625 | -1.019 | 0.308 | -1.356 | 0.373 | -0.690 | 0.924 |
| **dog_typesheperdtype** | -5.471 | 2.057 | 0.004 | -2.660 | 0.008 | -9.468 | -1.750 | -6.880 | -4.012 |
| **age** | 0.780 | 0.374 | 2.181 | 2.085 | 0.037 | 0.067 | 1.501 | 0.470 | 1.008 |
| **sexmale** | -2.913 | 2.062 | 0.054 | -1.412 | 0.158 | -6.843 | 1.257 | -4.004 | -1.188 |

Table 30 - WHINE

|  | **Nested models** | **df** | **AIC** | **-2LL** | **Likelihood ratio test** |
| --- | --- | --- | --- | --- | --- |
| **WHINE** | **Null** | 10 | 293.587 | 273.587 | -- |
|  | **Plus pre-cortisol** | 11 | 295.551 | 273.551 | χ^2^_2_= 0.035, p = .851 |
| as.data.frame(anova(full.WHINE, null.WHINE, test= “Chisq”) | | | | | |

| **WHINE - DURATION** | **Estimate** | **Std. Error** | **OR** | **z-value** | **P-value** | **Lower Ci** | **Upper CI** | **min** | **max** |
| --- | --- | --- | --- | --- | --- | --- | --- | --- | --- |
| **(Intercept)** | 0.298 | 0.263 |  |  |  | -0.208 | 0.797 | 0.166 | 0.388 |
| **pre_c** | -0.015 | 0.082 | 0.985 | -0.188 | 0.851 | -0.177 | 0.145 | -0.124 | 0.052 |
| **conditionFS** | 0.177 | 0.166 | 1.193 | 1.067 | 0.286 | -0.163 | 0.475 | -0.016 | 0.266 |
| **conditionPA** | -0.070 | 0.165 | 0.932 | -0.427 | 0.669 | -0.386 | 0.245 | -0.164 | 0.055 |
| **post_c** | -0.024 | 0.057 | 0.976 | -0.426 | 0.670 | -0.132 | 0.088 | -0.052 | 0.027 |
| **dog_typesheperdtype** | 0.096 | 0.202 | 1.100 | 0.479 | 0.632 | -0.308 | 0.505 | -0.005 | 0.171 |
| **age** | -0.032 | 0.036 | 0.969 | -0.879 | 0.380 | -0.107 | 0.037 | -0.042 | -0.007 |
| **sexmale** | 0.405 | 0.203 | 1.499 | 1.991 | 0.046 | 0.016 | 0.784 | 0.248 | 0.545 |

Table 31 - PUSHING THE APPARATUS

|  | **Nested models** | **df** | **AIC** | **-2LL** | **Likelihood ratio test** |
| --- | --- | --- | --- | --- | --- |
| **PUSHING APPARATUS** | **Null** | 10 | 454.257 | 434.257 | -- |
|  | **Plus pre-cortisol** | 11 | 454.230 | 432.230 | χ^2^_2_= 2-027, p = .155 |
| as.data.frame(anova(full.PUSHING, null.PUSHING, test= “Chisq”) | | | | | |

| **PUSHING APPARATUS - DURATION** | **Estimate** | **Std. Error** | **OR** | **z-value** | **P-value** | **Lower CI** | **Upper CI** | **min** | **max** |
| --- | --- | --- | --- | --- | --- | --- | --- | --- | --- |
| **(Intercept)** | 0.076 | 0.656 |  |  |  | 0.512 | 1.846 | -0.414 | 0.419 |
| **pre_c** | 0.247 | 0.172 | 1.280 | 1.435 | 0.151 | -0.177 | 0.101 | 0.005 | 0.381 |
| **conditionFS** | 0.065 | 0.291 | 1.067 | 0.222 | 0.824 | -0.729 | 0.187 | -0.030 | 0.179 |
| **conditionPA** | 0.576 | 0.290 | 1.778 | 1.990 | 0.047 | -0.521 | 0.225 | 0.058 | 0.780 |
| **post_c** | -0.037 | 0.117 | 0.964 | -0.316 | 0.752 | -0.083 | 0.088 | -0.078 | 0.044 |
| **dog_typesheperdtype** | -0.084 | 0.523 | 0.010 | -0.161 | 0.872 | -0.520 | 0.222 | -0.458 | 0.519 |
| **age** | 0.179 | 0.095 | 1.196 | 1.887 | 0.059 | -0.037 | 0.039 | 0.111 | 0.247 |
| **sexmale** | -1.146 | 0.526 | 0.318 | -2.180 | 0.029 | -0.318 | 0.311 | -1.575 | -0.428 |

Table 32 - SNIFFING THE ENVIRONMENT

|  | **Nested models** | **df** | **AIC** | **-2LL** | **Likelihood ratio test** |
| --- | --- | --- | --- | --- | --- |
| **SNIFFING ENVIRONMENT** | **Null** | 10 | 431.962 | 411.962 | -- |
|  | **Plus pre-cortisol** | 11 | 431.962 | 410.954 | χ^2^_2_= 1.008, p = .315 |
| as.data.frame(anova(full.SNIFFING, null.SNIFFING, test= “Chisq”) | | | | | |

| **SNIFFING ENVIRONMENT - DURATION** | **Estimate** | **Std. Error** | **OR** | **z-value** | **P-value** | **Lower CI** | **Upper  CI** | **min** | **max** |
| --- | --- | --- | --- | --- | --- | --- | --- | --- | --- |
| **(Intercept)** | 0.173 | 0.464 |  |  |  | -0.000 | 1.092 | -0.105 | 0.342 |
| **pre_c** | 0.146 | 0.145 | 1.157 | 1.006 | 0.314 | -0.075 | 0.138 | -0.065 | 0.198 |
| **conditionFS** | -0.774 | 0.328 | 0.461 | -2.360 | 0.018 | -0.468 | 0.274 | -0.962 | -0.357 |
| **conditionPA** | -0.776 | 0.326 | 0.460 | -2.378 | 0.017 | -0.415 | 0.142 | -0.966 | -0.500 |
| **post_c** | 0.322 | 0.100 | 1.379 | 3.238 | 0.001 | -0.081 | 0.076 | 0.256 | 0.432 |
| **dog_typesheperdtype** | 0.492 | 0.300 | 1.635 | 1.640 | 0.101 | -0.097 | 0.433 | 0.148 | 0.649 |
| **age** | -0.017 | 0.064 | 0.983 | -0.273 | 0.785 | -0.027 | 0.037 | -0.041 | 0.022 |
| **sexmale** | -0.121 | 0.349 | 0.886 | -0.347 | 0.728 | -0.376 | 0.154 | -0.386 | 0.117 |
